# Plasma microrna expression profile for reduced ejection fraction in dilated cardiomyopathy

**DOI:** 10.1038/s41598-021-87086-1

**Published:** 2021-04-06

**Authors:** Maria Calderon-Dominguez, Thalía Belmonte, Maribel Quezada-Feijoo, Mónica Ramos, Juan Calderon-Dominguez, Oscar Campuzano, Alipio Mangas, Rocio Toro

**Affiliations:** 1 Biomedical Research and Innovation Institute of Cadiz (INiBICA), Research Unit, Puerta del Mar University Hospital, Av/Ana de Viya 21, 11009 Cadiz, Spain; 2 Cardiology Department, Cruz Roja Hospital, Madrid, Spain; 3 Universidad Alfonso X, Madrid, Spain; 4 Cardiovascular Genetics Center, University of Girona-IDIBGI, Girona, Spain; 5 Medical Science Department, School of Medicine, University of Girona, Girona, Spain; 6 Centro de Investigación Biomédica en Red, Enfermedades Cardiovasculares (CIBERCV), Madrid, Spain; 7 Internal Medicine Department, Puerta del Mar University Hospital, School of Medicine, University of Cadiz, Cadiz, Spain; 8 Medicine Department, School of Medicine, University of Cadiz, Edifício Andrés Segovia 3º Floor, C/Dr Marañón S/N, 21001 Cádiz, Spain

**Keywords:** Biomarkers, Cardiology, Diseases, Medical research, Molecular medicine

## Abstract

The left ventricular (LV) ejection fraction (EF) is key to prognosis in dilated cardiomyopathy (DCM). Circulating microRNAs have emerged as reliable biomarkers for heart diseases, included DCM. Clinicians need improved tools for greater clarification of DCM EF categorization, to identify high-risk patients. Thus, we investigated whether microRNA profiles can categorize DCM patients based on their EF. 179-differentially expressed circulating microRNAs were screened in two groups: (1) non-idiopathic DCM; (2) idiopathic DCM. Then, 26 microRNAs were identified and validated in the plasma of ischemic-DCM (n = 60), idiopathic-DCM (n = 55) and healthy individuals (n = 44). We identified fourteen microRNAs associated with echocardiographic variables that differentiated idiopathic DCM according to the EF degree. A predictive model of a three-microRNA (miR-130b-3p, miR-150-5p and miR-210-3p) combined with clinical variables (left bundle branch block, left ventricle end-systolic dimension, lower systolic blood pressure and smoking habit) was obtained for idiopathic DCM with a severely reduced-EF. The receiver operating characteristic curve analysis supported the discriminative potential of the diagnosis. Bioinformatics analysis revealed that miR-150-5p and miR-210-3p target genes might interact with each other with a high connectivity degree. In conclusion, our results revealed a three-microRNA signature combined with clinical variables that highly discriminate idiopathic DCM categorization. This is a potential novel prognostic biomarker with high clinical value.

## Introduction

Dilated cardiomyopathy (DCM) is a heterogeneous heart disease characterized by the presence of left ventricular (LV) dilatation and systolic dysfunction in the absence of abnormal loading conditions or coronary artery disease sufficient to cause global systolic impairment^1,2^. This entity encompasses different phenotypes, clinical features and etiologies with a common final pathway. The prevalence of DCM is estimated at more than one in 250 individuals and it has increased by 27% in the last ten years. Hence, DCM is a health burden worldwide that imposes huge economic healthcare concerns due to high hospitalization, morbidity and mortality rates^3^.

The latest guidelines have established that the systolic ejection fraction (EF) discerns between heart failure (HF) categories^4^. Left ventricular ejection fraction (LVEF) has been strongly associated with adverse cardiac outcome in DCM^5–7^. Severely depressed EF, below 35%, has been shown in randomized clinical trials as a sudden death risk factor^8^. Nevertheless, the need for expert staff and inaccessible or excessive radiation imaging techniques impede clinical follow-up. Furthermore, highly time-consuming imaging techniques restrict access to the LV systolic function in the DCM population. Circulating levels of B-type natriuretic peptide or related molecules are very useful for clinical tailoring. However, they are not highly specific, as they are not good as systolic function markers^4^. The lack of solid biomarkers and the limitations of imaging cardiovascular tests to categorize LVEF in DCM urge the investigation of a new, reliable tool.

MicroRNAs (miRNAs) are evolutionary conserved non-coding RNA molecules of 19–25 nucleotides that play crucial roles in modulating the expression of most proteins at post-transcriptional level^9^. Their properties make them the most widely studied extracellular RNAs as diagnostic, prognostic and therapeutic markers in the cardiovascular field^10–12^. Several studies have described the relationship between DCM and miRNA profiles^13–15^. Therefore, a deeper knowledge of miRNA and the LVEF subcategories in the DCM population is needed to identify high-risk patients.

In the current study, we evaluated plasma miRNAs as potential biomarkers to discriminate severe from moderate reduced EF in a DCM population.

## Results

### Demographics and clinical manifestations in the patients

The subject characteristics are shown in Table 1. The mean age for control, idiopathic, and ischemic DCM patients was 39.1 ± 12.8, 67.3 ± 9.2, and 68.2 ± 8.4 years old. Most of the patients enrolled were men. As expected, LVEF and LVESD differed significantly between DCM patients and control subjects (*p* < 0.0001, for both comparisons). DCM group exhibited an increase in left atrium diameter (LA) and volume, sphericity index, and E/e’ ratio, compared with the control group. Regarding DCM^MOD^ group, ischemic patients showed significantly reduced heart rate, LVEF and right ventricle diameter compared with idiopathic patients (*p* = 0.014, *p* = 0.020 and *p* = 0.024, respectively). As to the DCM^SEV^ group, 10 ischemic patients had *diabetes mellitus* type 2, while only 4 idiopathic patients showed this comorbidity (*p* = 0.01). In both cases, sphericity index was significantly reduced in ischemic patients (*p* = 0.047 for DCM^MOD^ group and *p* = 0.03 for DCM^SEV^ group).

**Table 1:** Baseline characteristics of of the study groups.

Variables	DCM^MOD^		*p*	DCM^SEV^		*p*	Healthy control
Etiology	Idiopathic	Ischemic		Idiopathic	Ischemic		
n	40	46		15	14		44
**Demographics**							
Age (years)	67.6 ± 9.4	68.0 ± 8.3	NS	66.5 ± 8.4	68.7 ± 8.4	NS	39.1 ± 12.8
Sex (male, %)	70.0	76.1	NS	60.0	92.9	0.045	52.3
SBP (mm Hg)	124.1 ± 21.0	125.0 ± 14.5	NS	113.8 ± 17.5	118.7 ± 11.3	NS	114.4 ± 8.6
DBP (mm Hg)	71.7 ± 6.8	72.2 ± 11.1	NS	73.1 ± 10.6	70.8 ± 9.8	NS	74.4 ± 8.1
Heart rate (b.p.m)	71.0 ± 13.3	65.1 ± 12.9	0.014	67.1 ± 5.4	68.0 ± 11.5	NS	63.7 ± 11.4
LBBB (%)	15	8.7	NS	33.3	14.3	NS	0
Body mass index (kg/m^2^)	29.4 ± 5.6	29.1 ± 4.4	NS	28.0 ± 5.3	27.5 ± 3.7	NS	24.0 ± 3.5
Diabetes Mellitus (%)	40	46.7	NS	26.7	71.4	0.019	0
Smoking habit (%)	40	47.8	NS	73	69.2	NS	0
II-III NYHA functional class (%)	12.5	10.9	NS	20	35.7	NS	0
**Medications**							
ACE I (%)	42.5	41.3	NS	60	35.7	NS	0
Antiplatelet (%)	37.5	30.4	NS	26.7	42.9	NS	0
ARA II (%)	33.3	41.3	NS	55	71.4	0.047	0
Beta-blocker (%)	65	71.7	NS	100	100	NS	0
Statin (%)	22.5	32.6	NS	33.3	28.6	NS	29.5
**Echocardiography**							
LVEF (%)	39.0 ± 4.5	36.7 ± 4.0	0.020	24.1 ± 4.4	25.7 ± 3.6	NS	66.2 ± 5.2
LVEDD (mm)	63.1 ± 4.1	63.3 ± 6.2	NS	60.5 ± 5.4	60.4 ± 5.8	NS	46.2 ± 4.9
LVESD (mm)	45.8 ± 14.0	46.1 ± 13.5	NS	54.6 ± 5.4	42.9 ± 18.6	NS	28.4 ± 5.8
Right ventricle (mm)	37 ± 8.5	33.4 ± 7.3	0.024	37.9 ± 11.7	34.9 ± 7.2	NS	27.2 ± 4.0
MAPSE	11 ± 2.3	10.9 ± 2.1	NS	1 ± 3.2	9.5 ± 2.0	NS	18.9 ± 2.2
TAPSE	20 ± 4.7	19.8 ± 4.7	NS	19. 2 ± 6.3	19.5 ± 3.6	NS	21.4 ± 3.3
Aortic root (mm)	33.9 ± 5.0	33.2 ± 4.8	NS	30. 3 ± 6.2	32.2 ± 3.5	NS	–
E/e’ ratio	15.2 ± 6.8	15.1 ± 6.5	NS	15.2 ± 8.1	16.9 ± 8.7	NS	7.5 ± 1.7
Left Atrium (mm)	46.7 ± 9.2	44.3 ± 7.7	NS	41.1 ± 8.2	45.1 ± 7.3	NS	35.8 ± 8.3
Sphericity index	0.8 ± 0.3	0.7 ± 0.1	0.047	0.9 ± 0.27	0.7 ± 0.1	0.032	0.5 ± 0.1

Data presented as mean ± SD for continuous variables and as percentage for categorical variables. NS, no significant. Abbreviations: ACE I, angiotensin-converting enzyme; ARA II, angiotensin II receptor antagonist; b.p.m, beats per minute; DBP, diastolic blood pressure; DCM^MOD^, dilated cardiomyopathy with ejection fraction ≥ 31–49%; DCM^SEV^, dilated cardiomyopathy with ejection fraction ≤ 30%; E/e’, ratio of mitral early diastolic flow velocity over tissue Doppler lateral mitral annular lengthening velocity; LBBB, left bundle branch block; LVEDD, left ventricle end-diastolic diameter; LVEF, left ventricle ejection fraction; LVESD, left ventricle end-systolic diameter; MAPSE, mitral annular plane systolic excursion; NYHA, New York Heart Association; SBP, systolic blood pressure; TAPSE, tricuspid annular plane systolic excursion.

### Screening study

**Figure 1:**
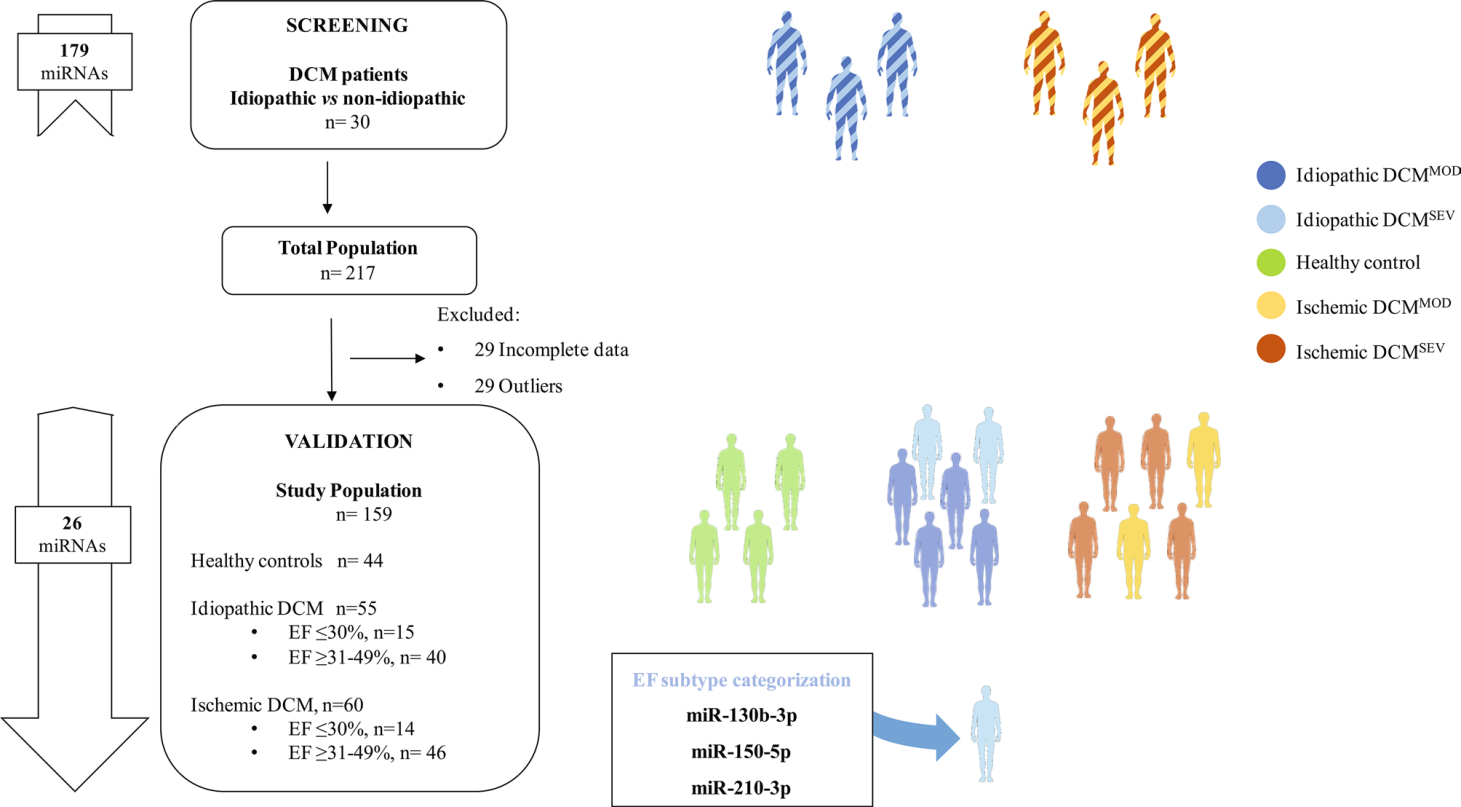
Flowchart of the study design strategy. This figure illustrates the experimental workflow of the study including screening, validation and EF status of DCM patients. Abbreviations: DCM, dilated cardiomyopathy; DCM^MOD^, dilated cardiomyopathy with ejection fraction ≥ 31–49%; DCM^SEV^, dilated cardiomyopathy with ejection fraction ≤ 30%; EF, ejection fraction.

The study design is shown in Fig. 1. We first screened 179-differentially expressed circulating miRNAs in 30 idiopathic and non-idiopathic DCM age- and sex-matched patients (see Table S1 and Fig. S1 in Supplementary Material). In total, 26 miRNAs were significantly expressed according to the selection criteria at high levels and statistical significance (median Cq < 32 and detected in at least 80% of all samples and *p* < 0.05). The candidates selected for further analysis were let-7 g-5p, let-7a-5p, miR-1-3p, miR-16-5p, miR-16–2-3p, miR-19b-3p, miR-25-3p, miR-29a-3p, miR-30b-5p, miR-30e-3p, miR-130b-3p, miR-133a-3p, miR-133b, miR-142-3p, miR-145-5p, miR-150-5p, miR-192-5p, miR-199a-3p, miR-210-3p, miR-215-5p, miR-324-3p, miR-363-3p, miR-454-3p, miR-532-5p, miR-629-5p and miR-660-5p (Table 2).

**Table 2:** Peripheral microRNA levels in the Idiopathic and Ischemic DCM *vs.* healthy control.

microRNA	Healthy control		Idiopathic DCM			Ischemic DCM		
	n	median (Q1-Q3)	n	median (Q1-Q3)	*p* value	n	median (Q1-Q3)	*p* value
let-7a-5p	44	6.543 (6.308–6.832)	55	6.511 (6.308–7.207)	0.6636	60	7.155 (6.507–7.626)	0.0001
let-7 g-5p	44	6.268 (6.095–6.477)	55	6.303 (6.095–6.939)	0.3767	60	6.555 (6.147–7.034)	0.0236
miR-1-3p	44	4.101 (3.878–4.460)	55	4.238 (3.878–5.059)	0.0611	60	4.491 (3.552–5.038)	0.1142
miR-16-5p	44	7.634 (7.379–7.900)	55	7.658 (7.379–8.304)	0.0046	60	8.025 (7.714–8.300)	0.0017
miR-16–2-3p	44	4.967 (4.706–5.281)	55	7.035 (6.773–5.609)	0.0043	60	5.363 (5.003–5.573)	0.0026
miR-19b-3p	44	7.014 (6.773–7.230)	55	6.542 (6.199–7.637)	0.008	60	7.517 (7.103–7.751)	0.0006
miR-25-3p	44	6.487 (6.199–6.751)	55	6.230 (6.052–7.178)	0.0011	60	6.917 (6.620–7.127)	0.0017
miR-29a-3p	44	5.480 (5.297–5.643)	55	4.951 (4.730–6.233)	0.0001	60	5.951 (5.630–6.304)	0.0001
miR-30b-5p	44	6.197 (6.052–6.431)	55	4.913 (4.745–6.739)	0.9142	60	6.525 (6.032–6.992)	0.0229
miR-30e-3p	44	4.923 (4.745–5.165)	55	5.072 (4.858–5.559)	0.145	60	5.346 (4.793–5.722)	0.0127
miR-130b-3p	44	5.058 (4.858–5.275)	55	4.483 (4.167–5.849)	0.0001	60	5.610 (5.056–5.904)	0.0002
miR-133a-3p	44	4.451 (4.167–4.741)	55	4.164 (3.843–5.412)	0.0001	60	4.873 (4.273–5.439)	0.0042
miR-133b	44	4.129 (3.843–4.437)	55	6.950 (6.690–5.028)	0.0019	60	4.491 (3.909–5.105)	0.0026
miR-142-3p	44	6.932 (6.690–7.217)	55	5.454 (5.279–7.588)	0.9753	60	7.425 (7.007–8.022)	0.0001
miR-145-5p	44	5.423 (5.279–5.629)	55	5.504 (5.313–6.044)	0.2254	60	5.867 (5.473–6.385)	0.0001
miR-150-5p	44	5.520 (5.313–5.696)	55	4.970 (4.665–6.133)	0.1293	60	5.803 (5.377–6.053)	0.0634
miR-192-5p	44	4.928 (4.665–5.152)	55	6.231 (6.110–5.607)	0.0708	60	5.293 (4.893–5.486)	0.0075
miR-199a-3p	44	6.218 (6.110–6.390)	55	4.512 (4.314–6.967)	0.0007	60	6.628 (6.018–7.044)	0.0047
miR-210-3p	44	4.514 (4.314–4.705)	55	4.809 (4.544–5.317)	0.0001	60	5.039 (4.683–5.478)	0.0001
miR-215-3p	44	4.770 (4.544–4.997)	55	5.536 (5.299–5.397)	0.0479	60	5.101 (4.720–5.397)	0.0098
miR-324-3p	44	4.892 (4.728–5.139)	55	5.318 (4.969–5.554)	0.0079	60	5.397 (4.957–5.689)	0.0002
miR-363-3p	44	5.260 (4.969–5.555)	55	3.845 (3.281–5.946)	0.002	60	5.750 (5.389–5.893)	0.0006
miR-454-3p	44	3.759 (3.281–4.098)	55	4.704 (4.454–4.437)	0.7829	60	4.130 (3.298–4.699)	0.0061
miR-532-5p	44	4.668 (4.454–4.854)	55	5.535 (5.297–5.344)	0.083	60	5.207 (4.782–5.398)	0.0002
miR-629-5p	44	4.332 (4.104–4.680)	55	5.281 (4.854–5.72)	< 0.0001	60	5.182 (4.769–5.813)	< 0.0001
miR-660-5p	44	4.936 (4.730–5.162)	55	5.436 (5.104–5.639)	0.0001	60	5.454 (5.092–5.614)	0.0001

Data presented as median (Q1-Q3). Samples size is indicated. Abbreviations: DCM. dilated cardiomyopathy.

### Validation study and association with a population that has a reduced ejection fraction and DCM

The ischemic and idiopathic DCM populations are presented as DCM^MOD^ and DCM^SEV^ groups based on their LVEF, ≤ 30% and > 31–49%, respectively. We investigated the potential value of the miRNA candidates to discriminate between the DCM^SEV^ *vs*. the DCM^MOD^ population and their association with echocardiographic parameters (Table 3 and Table S2). Only the idiopathic DCM^SEV^ group showed an association between LVEF and fourteen miRNA candidates (Table 4). These miRNAs were let-7a-5p, let 7 g-5p, miR-19b-3p, miR-25-3p, miR-29a-3p, miR-30b-5p, miR-30e-3p, miR-142-3p, miR-145-5p, miR-150-5p, miR-199a-3p, miR-210-3p, , miR-324-3p and miR-660-5p. No association was found in the idiopathic DCM^MOD^ cohort with LVEF (Table 4). Moreover, the correlation between other echocardiographic variables and individual miRNAs was also identified. All these miRNAs significantly correlated with LVEF in DCM^SEV^ patients. In addition, these miRNAs in the DCM^MOD^ group were significantly correlated with LV end-systolic dimension (LVESD) and the left atrium dimension (LA).

**Table 3:** Peripheral microRNA levels for LVEF categorization in idiopathic and ischemic DCM.

microRNA	Idiopathic DCM			Ischemic DCM		
	DCM^SEV^	DCM^MOD^	*p value*	DCM^SEV^	DCM^MOD^	*p value*
	median (Q1-Q3)	median (Q1-Q3)		median (Q1-Q3)	median (Q1-Q3)	
let-7a-5p	6.460 (5.732–7.080)	6.909 (6.059–7.226)	0.009	7.206 (6.547–7.594)	7.125 (6.342–7.707)	0.893
let-7 g-5p	6.272 (5.894–6.482)	6.668 (6.000–7.010)	0.003	6.712 (5.940–6.993)	6.548 (6.138–7.054)	0.997
miR-1-3p	4.772 (4.493–5.241)	4.398 (2.779–4.950)	0.003	4.732 (2.779–5.128)	4.432 (3.242–4.989)	0.995
miR-16-5p	8.127 (7.898–8.513)	7.900 (7.619–8.221)	0.552	7.930 (7.425–8.251)	8.040 (7.681–8.301)	0.997
miR-16–2-3p	5.386 (5.132–5.694)	5.233 (5.016–5.559)	0.651	5.338 (4.857–5.550)	5.381 (4.994–5.588)	0.999
miR-19b-3p	7.218 (6.949–7.581)	7.449 (7.112–7.637)	0.044	7.486 (6.814–7.671)	7.539 (7.100–7.775)	0.997
miR-25-3p	6.697 (6.293–7.524)	6.938 (6.638–7.175)	0.007	6.858 (6.320–7.047)	6.919 (6.577–7.153)	0.996
miR-29a-3p	5.746 (5.576–6.095)	5.973 (5.718–6.258)	0.0326	6.077 (5.176–6.289)	5.949 (5.635–6.329)	0.825
miR-30b-5p	5.880 (5.498–6.397)	6.488 (5.634–6.862)	0.011	6.684 (5.846–6.923)	6.468 (5.982–7.027)	0.946
miR-30e-3p	4.926 (4.563–5.320)	5.371 (4.697–5.622)	0.001	5.507 (4.551–5.699)	5.186 (4.779–5.781)	0.952
miR-130b-3p	5.653 (5.371–5.923)	5.493 (5.236–5.846)	0.956	5.625 (4.955–5.872)	5.542 (5.048–5.920)	0.834
miR-133a-3p	5.197 (4.906–5.412)	4.895 (4.429–5.433)	0.365	4.890 (3.212–5.439)	4.851 (4.269–5.429)	0.968
miR-133b	4.784 (4.459–5.196)	4.434 (3.899–4.994)	0.193	4.689 (3.204–5.061)	4.485 (3.890–5.111)	0.760
miR-142-3p	6.770 (5.961–7.348)	7.233 (6.286–7.600)	0.0005	7.749 (6.859–8.046)	7.384 (6.741–8.023)	0.340
miR-145-5p	5.389 (5.113–5.758)	5.654 (5.089–6.126)	0.005	6.005 (5.466–6.175)	5.761 (5.221–6.408)	0.998
miR-150-5p	5.539 (5.143–6.016)	5.790 (5.418–6.156)	0.004	5.446 (5.031–5.806)	5.852 (5.451–6.066)	0.916
miR-192-5p	5.192 (5.107–5.614)	5.119 (4.857–5.579)	0.438	5.179 (4.712–5.523)	5.294 (4.827–5.508)	0.757
miR-199a-3p	6.527 (6.240–6.733)	6.715 (6.367–7.000)	0.036	6.906 (5.961–7.026)	6.544 (6.104–7.081)	0.962
miR-210-3p	4.781 (4.340–5.179)	5.058 (4.655–5.364)	0.003	5.023 (4.382–5.479)	5.040 (4.679–5.479)	0.797
miR-215-3p	5.158 (4.784–5.616)	4.993 (4.662–5.389)	0.773	5.084 (4.449–5.356)	5.110 (4.670–5.401)	0.874
miR-296a-3p	6.099 (5.849–6.409)	5.883 (5.590–6.168)	0.032	6.077 (5.176–6.289)	5.949 (5.635–6.329)	0.825
miR-324-3p	5.040 (4.827–5.385)	5.360 (4.960–5.570)	0.001	5.454 (4.695–5.689)	5.371 (4.945–5.697)	0.858
miR-363-3p	5.698 (5.512–6.065)	5.576 (5.255–5.928)	0.778	5.757 (5.094–5.908)	5.712 (5.349–5.896)	0.999
miR-454-3p	3.069 (3.069–4.437)	3.069 (3.069–4.438)	0.926	4.442 (3.654–4.635)	4.051 (3.069–4.758)	0.418
miR-532-5p	5.152 (4.748–5.622)	4.917 (4.459–5.315)	0.285	5.207 (4.468–5.407)	5.192 (4.741–5.403)	0.985
miR-629-5p	4.902 (4.651–5.566)	5.538 (5.112–5.903)	0.123	5.465 (5.306–5.918)	5.852 (5.451–5.065)	0.979
miR-660-5p	5.184 (5.049–5.899)	5.465 (5.196–5.570)	0.034	5.454 (4.608–5.697)	5.4504 (5.050–5.624)	0.992

Data presented as median (Q1-Q3). Samples size is indicated. Abbreviations: DCM. dilated cardiomyopathy; DCM^MOD^, dilated cardiomyopathy with ejection fraction ≥ 31–49%; DCM^SEV^, dilated cardiomyopathy with ejection fraction ≤ 30%.

**Table 4:** Correlation between the echocardiographic parameter and individual microRNAs for LVEF categorization in idiopathic DCM.

microRNA	LVEF (%)				LVESD (mm)				Left atrial dimension (mm)			
	DCM^SEV^		DCM^MOD^		DCM^SEV^		DCM^MOD^		DCM^SEV^		DCM^MOD^	
	Pearson r	*p*	Pearson r	*p*	Pearson r	*p*	Pearson r	*p*	Pearson r	*p*	Pearson r	*p*
Let-7a-5p	− 0.677	0.006	0.140	0.388	− 0.103	0.726	− 0.489	0.002	0.277	0.318	− 0.362	0.022
Let-7 g-5p	− 0.609	0.016	0.181	0.269	− 0.286	0.321	− 0.408	0.011	0.368	0.269	− 0.532	< 0.001
miR-1-3p	− 0.190	0.498	0.204	0.206	0.046	0.875	− 0.218	0.183	0.242	0.385	− 0.433	0.005
miR-16-5p	− 0.190	0.498	0.204	0.206	0.046	0.875	− 0.218	0.183	0.242	0.385	− 0.433	0.005
miR-16–2-3p	− 0.438	0.102	0.130	0.424	0.044	0.882	− 0.559	< 0.001	0.219	0.432	− 0.262	0.102
miR-19b-3p	− 0.527	0.043	0.193	0.233	0.111	0.705	− 0.444	0.005	0.276	0.319	− 0.306	0.054
miR-25-3p	− 0.538	0.039	0.106	0.515	0.077	0.795	− 0.544	< 0.001	0.155	0.580	− 0.246	0.127
miR-29a-3p	− 0.641	0.010	0.168	0.300	0.180	0.538	− 0.261	0.109	0.337	0.219	− 0.336	0.034
miR-30b-5p	− 0.603	0.017	0.179	0.269	− 0.312	0.278	− 0.376	0.018	0.313	0.256	− 0.436	0.005
miR-30e-3p	− 0.629	0.012	0.234	0.145	− 0.130	0.658	− 0.335	0.037	0.385	0.157	− 0.347	0.028
miR-130b-3p	− 0.394	0.146	0.094	0.564	0.109	0.710	− 0.296	0.067	0.554	0.032	− 0.269	0.093
miR-133a-3p	− 0.455	0.088	0.092	0.574	0.160	0.584	− 0.359	0.025	0.208	0.456	− 0.348	0.028
miR-133b	− 0.361	0.186	0.179	0.268	0.028	0.924	− 0.194	0.236	0.140	0.619	− 0.444	0.004
miR-142-3p	− 0.569	0.027	0.210	0.193	− 0.080	0.787	− 0.417	0.008	0.359	0.189	− 0.349	0.027
miR-145-5p	− 0.614	0.015	0.130	0.423	0.109	0.711	− 0.425	0.007	0.324	0.239	− 0.382	0.015
miR-150-5p	− 0.540	0.038	0.178	0.272	− 0.148	0.614	− 0.357	0.025	0.327	0.234	− 0.419	0.007
miR-192-5p	− 0.464	0.082	0.237	0.141	0.152	0.603	− 0.536	< 0.001	0.191	0.495	− 0.252	0.117
miR-199a-3p	− 0.692	0.004	0.184	0.257	0.065	0.826	− 0.198	0.228	0.396	0.144	− 0.401	0.010
miR-210-3p	− 0.555	0.032	0.188	0.244	0.056	0.848	− 0.405	0.011	0.285	0.304	− 0.287	0.073
miR-215-3p	− 0.425	0.114	0.201	0.213	− 0.003	0.993	− 0.583	0.000	0.109	0.699	− 0.301	0.059
miR-324-3p	− 0.667	0.007	0.183	0.257	− 0.053	0.857	− 0.448	0.004	0.270	0.330	− 0.293	0.066
miR-363-3p	− 0.463	0.082	0.075	0.644	0.070	0.811	− 0.582	< 0.001	0.125	0.657	− 0.189	0.244
miR-454-3p	− 0.692	0.004	0.085	0.603	− 0.290	0.315	− 0.293	0.070	0.250	0.368	− 0.416	0.008
miR-532-5p	− 0.480	0.070	0.283	0.077	− 0.058	0.844	− 0.487	0.002	0.217	0.438	− 0.308	0.053
miR-629-5p	− 0.383	0.159	− 0.057	0.729	0.017	0.954	− 0.419	0.008	0.151	0.592	− 0.057	0.728
miR-660-5p	− 0.500	0.058	0.102	0.530	0.313	0.276	− 0.538	< 0.001	0.231	0.407	− 0.118	0.467

Abbreviations: DCM^MOD^, dilated cardiomyopathy with ejection fraction ≥ 31–49%; DCM^SEV^, dilated cardiomyopathy with ejection fraction ≤ 30%; LVEF, left ventricle ejection fraction; LVESD, left ventricle end-systolic diameter. Coefficient significant at *p* < 0.05.

**Figure 2:**
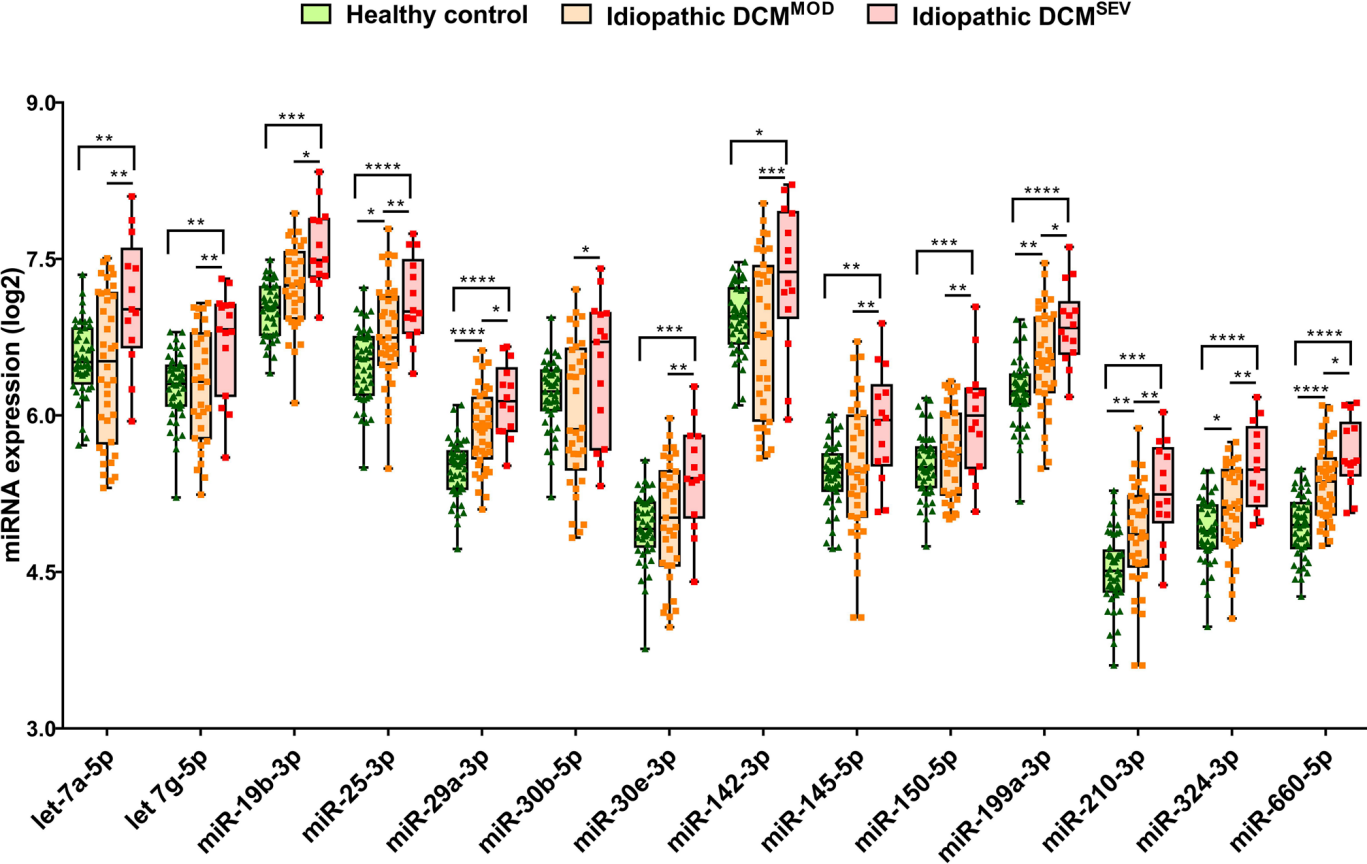
Boxplots of miRNA expression levels, normalized to cel-miR-39-3p, in healthy subjects, idiopathic DCM^MOD^ and DCM^SEV^. The analysis was carried out using qPCR. Data are presented in log_2_. Data represent the mean ± SEM. *p < 0.05, **p < 0.01, ***p < 0.001. Abbreviations: DCM^MOD^, dilated cardiomyopathy with ejection fraction ≥ 31–49%; DCM^SEV^, dilated cardiomyopathy with ejection fraction ≤ 30%.

**Figure 3:**
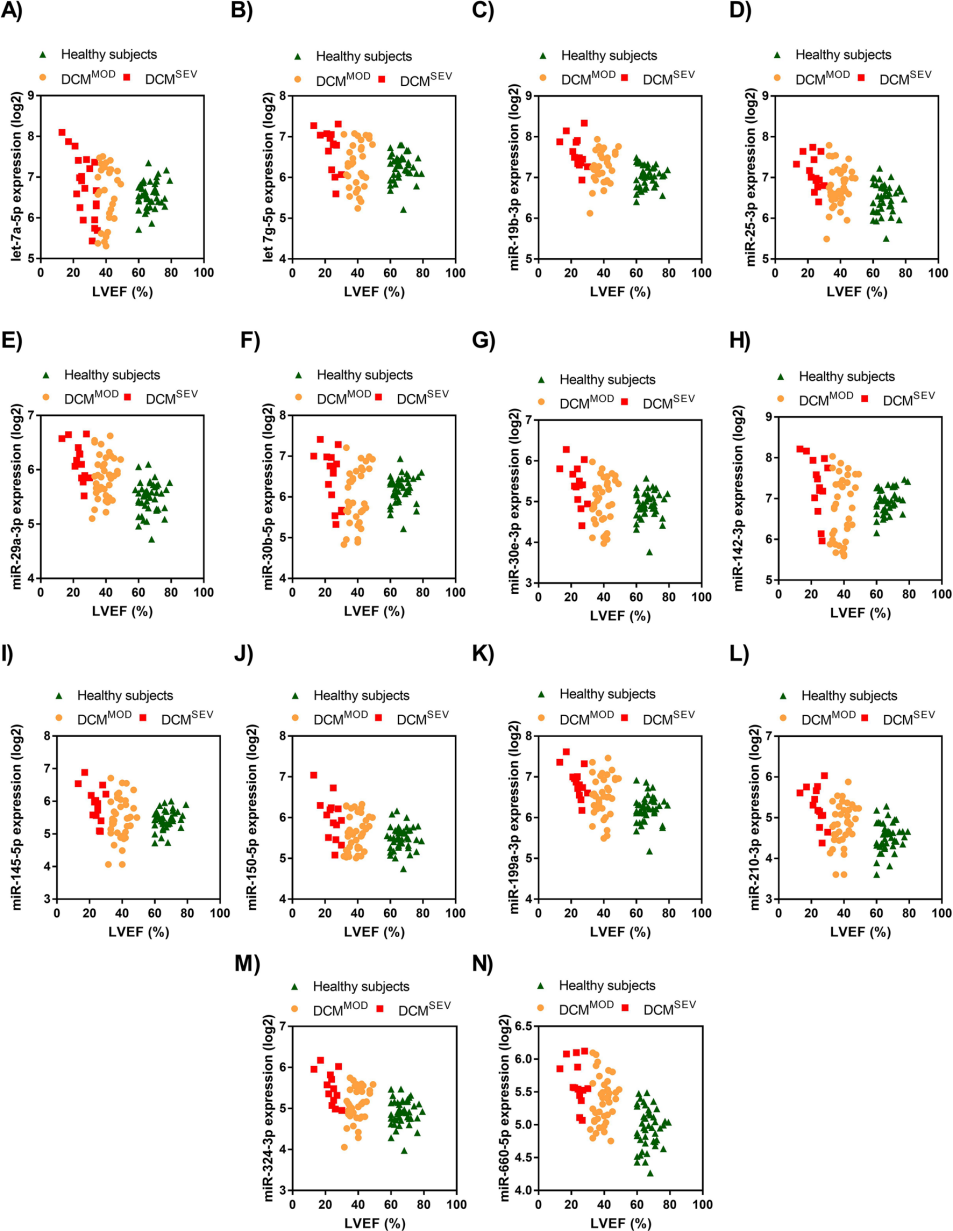
Correlation between the LVEF parameter and individual microRNAs for LVEF categorization in idiopathic DCM. (A-N) LVEF was independently negatively related with let-7a-5p (**A**), let 7 g-5p (**B**), miR-19b-3p (**C**), miR-25-3p (**D**), miR-29a-3p (**E**), miR-30b-5p (**F**), miR-30e-3p (**G**), miR-142-3p (**H**), miR-145-5p (**I**), miR-150-5p (**J**), miR-199a-3p (**K**), miR-210-3p (**L**), , miR-324-3p (**M**) and miR-660-5p (**N)** levels. Abbreviations: DCM^MOD^, dilated cardiomyopathy with ejection fraction ≥ 31–49%; DCM^SEV^, dilated cardiomyopathy with ejection fraction ≤ 30%.

A graphical illustration of the potential miRNA expression levels to discriminate between DCM^SEV^ and DCM^MOD^ in the idiopathic group is represented in Figs. 2 and 3. In all cases, the average miRNA levels were significantly higher in the idiopathic DCM group than in healthy subjects. Moreover, we found a significant increase in these circulating miRNA levels in plasma from DCM^SEV^ compared with DCM^MOD^ idiopathic patients.

### Combining miRNAs for DCM detection and subtype categorization

The ability of these miRNAs to discriminate between DCM with moderate and severe EF impairment was assessed using AUC-ROC. All individual miRNAs show an AUC ≥ 0.7, except for miR-30b-5p, miR-210-3p and miR-324-3p that were 0.69, 0.68 and 0.69, respectively. The highest AUC values achieved by single miRNAs was obtained for miR-145-5p with an AUC of 0.78 (95% CI: 0.65–0.91; *p* = 0.0029). The AUC values obtained for let-7a-5p, let 7 g-5p, miR-19b-3p, miR-25-3p, miR-29a-3p, miR-30e-3p, miR-142-3p, miR-150-5p, miR-199a-3p, and miR-660-5p when DCM^SEV^ were compared to DCM^MOD^ in the idiopathic group, as well as sensitivity for a whole range of specificities, are shown in Table 5.

**Table 5:** Comparisons of single microRNAs as predictors of idiopathic DCM^SEV^ from DCM^MOD^ and their diagnostic value.

miRNA	AUC (95% CI)	Sensitivity (%)	Specificity (%)	*p*
Let-7a-5p	0.73 (0.57–0.88)	81.58 68.42 63.16	38.46 46.15 61.54	0.0155
Let-7 g-5p	0.71 (0.55–0.86)	75.68 72.97 64.86	46.67 60 66.67	0.0213
miR-19b-3p	0.77 (0.62–0.92)	76.67 66.67 63.33	50 57.14 78.57	0.0050
miR-25-3p	0.70 (0.55–0.86)	78.95 68.42 60.53	35.71 57.14 64.29	0.0259
miR-29a-3p	0.70 (0.55–0.86)	72.5 70 52.5	50 57.14 71.43	0.0244
miR-30b-5p	0.69 (0.52–0.86)	82.35 73.53 55.88	46.67 53.33 60	0.0354
miR-30e-3p	0.72 (0.57–0.87)	78.38 72.97 70.27	35.71 42.86 64.29	0.0161
miR-142-3p	0.76 (0.62–0.91)	75.86 72.41 65.52	46.67 60 73.33	0.0046
miR-145-5p	0.78 (0.65–0.91)	78.95 73.68 65.79	46.15 61.54 69.23	0.0029
miR-150-5p	0.73 (0.58–0.89)	84.62 79.49 69.23	38.46 46.15 69.23	0.0119
miR-199a-3p	0.72 (0.58–0.87)	71.05 68.42 63.16	57.14 64.29 71.43	0.0145
miR-210-3p	0.68 (0.52–0.85)	81.58 68.42 63.16	40 53.33 66.67	0.0382
miR-324-3p	0.69 (0.52–0.86)	85.71 77.14 60	35.71 42.86 64.29	0.0417
miR-660-5p	0.73 (0.58–0.87)	75 70 60	57.14 64.29 71.43	0.0129

Abbreviations: AUC, area under the curve; CI, confidence interval; DCM^SEV^, dilated cardiomyopathy with ejection fraction ≤ 30%; miRNA, microRNA; ROC, receiver operating characteristic.

### A predictive model to determinate the association of microRNAs with a very reduced ejection fraction in idiopathic DCM

Statistical modelling was used to determine the association of the fourteen miRNA candidates with the patient characteristics. A multivariate model was built up with three predictive circulating miRNAs, miR-130b-3p, miR-150-5p and miR-210-3p. The multivariate logistic regression analyses revealed that circulating miR-130b-3p, miR-150-5p and miR-210-3p levels were associated with left bundle branch block (LBBB), LVESD, lower systolic blood pressure (SBP) and smoking habit in our idiopathic DCM^SEV^ group. Based on this analysis, LBBB (OR: 18.13; 95% CI: 1.28- 257.21; *p* = 0.014), LVESD (OR: 1.24; 95% CI: 1.01–1.53; *p* = 0.01), SBP (OR: 0.97; 95% CI: 0.91–1.03; *p* = 0.329), smoking habit (OR: 01; 95% CI: 1.44–630.02; *p* = 0.007), plasma miR-130b-3p (OR: 0; 95% CI: 0.1–1.57; *p* = 0.01), miR-150-5p (OR: 36.06; 95% CI: 1.64–794.78; *p* = 0.006) and miR-210-3p (OR: 687.53; 95% CI: 0.9–527,268.21; *p* < 0.001) were independent influencing factors for idiopathic DCM^SEV^ (Table 6). In addition, the Akaike Information Criterion (AIC) of our model showed a value of 35.406. The AIC for a model just with a combination of miR-150-5p and miR-210-3p levels showed a higher value (AIC: 61.393). This lower value indicates that our model has a better fit.

**Table 6:** Multivariate logistic regression analysis for identification of independent predictors of idiopathic DCM^SEV^ patients.

Variable	OR	95% CI	*p*
LBBB (%)	18.13	1.28–257.21	0.014
LVESD (mm)	1.24	1.01–1.53	0.01
SBP (mm Hg)	0.97	0.91–1.03	0.329
Smoking habit	30.1	1.44–630.02	0.007
miR-130b-3p	0	0.1–1.57	0.01
miR-150-5p	36.06	1.64–794.78	0.006
miR-210-3p	687.53	0.9–527,268.21	< 0.001

AIC value: 35.406.

The model included LBBB (%), LVESD (mm), SBP (mm Hg), smoking habit and the levels of miR-130b-3p,miR-150-5p and miR-210-3p. Abbreviations: AIC, Aikaike Information Criterion; CI, confidence interval; LBBB, left bundle branch block; LVESD, left ventricular end-systolic diameter; OR, odds ratio; SBP, systolic blood pressure.

**Figure 4:**
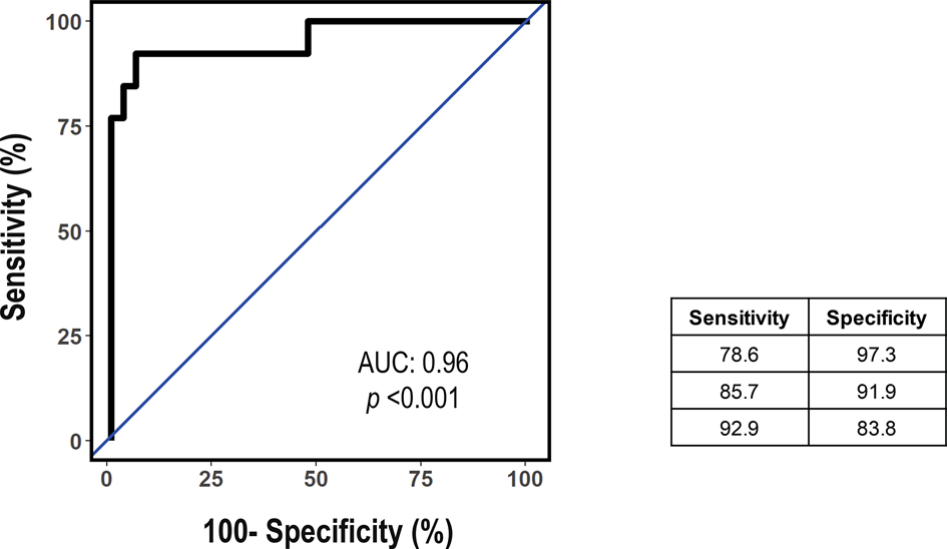
ROC analysis for the multivariate logistic regression model predicting idiopathic DCM^SEV^ patients. The mean AUC for the averaged ROC curves are presented. The data are presented as the AUC and 95% CI and corresponding sensitivity for a range of specificities. The AUC is 0.96 (95% CI: 0.884–1.00; *p* < 0.001). Abbreviations: AUC, area under the curve; CI, confidence interval; DCM^SEV^, dilated cardiomyopathy with ejection fraction ≤ 30%; ROC, receiver operating characteristic.

The potential usefulness of our model for identifying the idiopathic DCM^SEV^ population was obtained with a ROC curve analysis. As shown in Fig. 4, the AUC was 0.96 (95% CI: 0.884–1.00; *p* < 0.001).

### miRNA-gene network analysis

**Figure 5:**
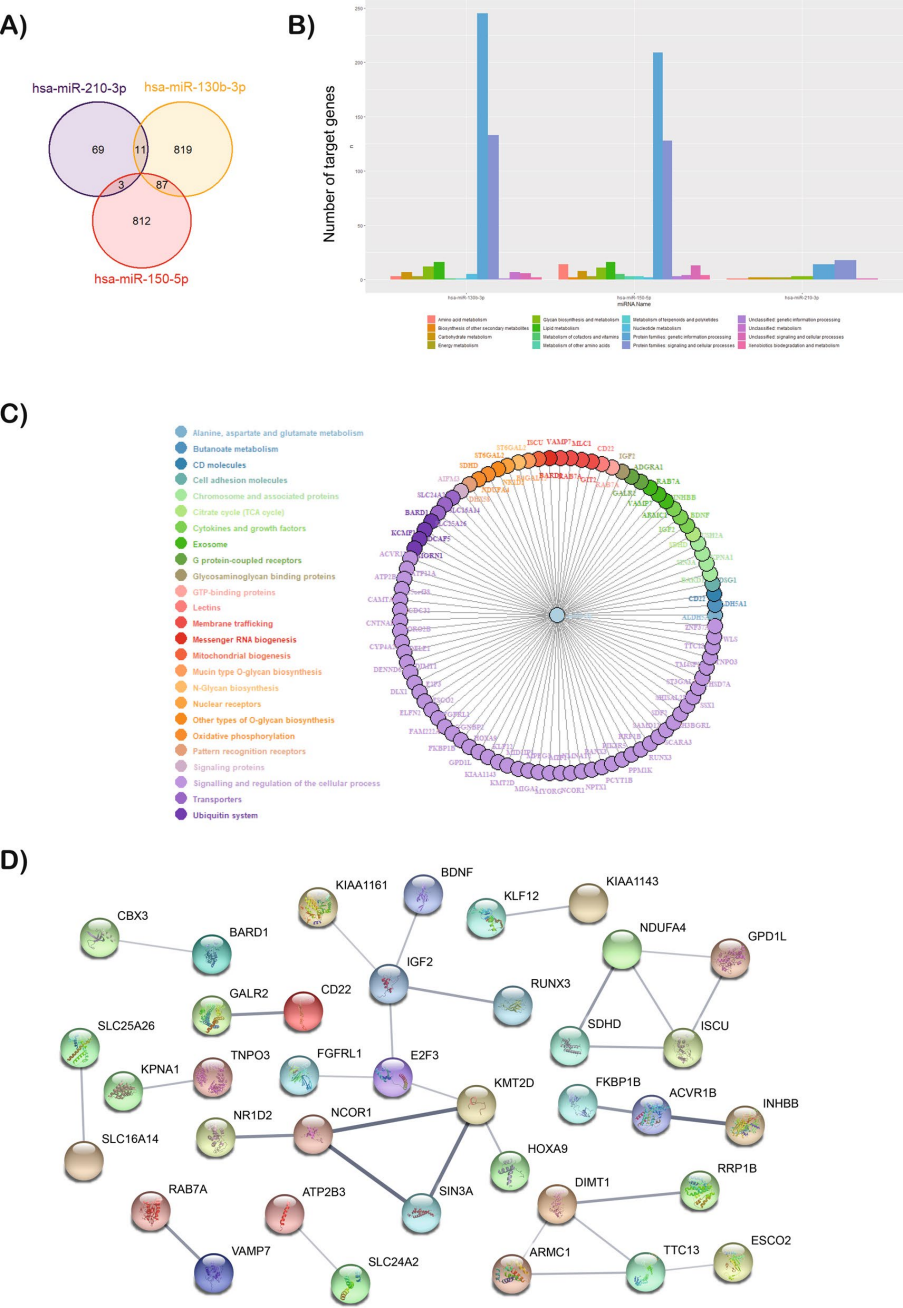
The miRNA-gene network for miRNAs associated with genes related to idiopathic DCM^SEV^. (**A**) Venn diagram showing overlap of gene targets of miR-130b-3p, miR-150-5p and miR-210-3p. (**B**) GO and KEGG analysis of miR-130b-3p, miR-150-5p and miR-210-3p target genes; the x-axis indicates KEGG pathway categories and the y-axis indicates the target numbers of genes. (**C**) miRNA-gene network for miR-210-3p. The network was built using 83 targeted genes and predicted interactions from the miRDB database. (**D**) PPI analysis performed using the STRING database for the target genes of miR-210-3p. The disconnected nodules are hidden. Bold lines indicate stronger association. Abbreviations: DCM^SEV^, dilated cardiomyopathy with ejection fraction ≤ 30%.

The hypothetical functions of three miRNAs were assessed with the miRDB database to reach the target mRNAs. The Gene Ontology (GO) and Kyoto Encyclopedia of Genes and Genomes (KEGG) pathways database identified the biological and molecular processes. The miR-130b-3p, miR-150-5p and miR-210-3p were associated with 917, 902 and 83 mRNAs in the miRDB database, respectively (Fig. 5A). The analysis revealed that most of these genes are involved in signalling and regulation of the cellular and metabolic process and genetic information processing (Figs. 5B–C). For instance, the genes are deeply involved in the transcription pathway since these miRNAs regulate the expression of the RNA Polymerase II, transcription factors or alternative splicing. In addition, other target genes are implicated in the nitrogen compound metabolic process or in response to oxygen levels, among others (extended in Fig. S3).

On the other hand, the protein–protein interaction networks (PPI) analysis networks related to miR-210-3p were analysed using the search tool for the retrieval of interacting genes (STRING) (Fig. 5D). Our *in-silico* model showed that ISCU and other mitochondrial activity-related proteins, as well as SDHD and NDUFA4, are targets of miR-210-3p. Moreover, the analysis revealed that genes targeted by miR-150 and miR-210 showed a significant PPI enrichment (*p* = 0.00459) (Figure S4 and Table S3). These results demonstrated that target genes of miR-210-3p and miR-150-5p may interact with each other.

## Discussion

We analysed the circulating miRNA signature of the ischemic and idiopathic DCM population stratified in severely and moderately reduced EF, ≤ 30% and 31–49% respectively. Our study reports, for the first time, a signature of three circulating miRNAs (miR-130b-3p, miR-150-5p and miR-210-3p) that allow us to discriminate between idiopathic DCM^MOD^ and DCM^SEV^ subjects. Furthermore, this circulating miRNA panel in plasma defines idiopathic DCM^SEV^ from ischemic etiology.

LVEF is the cornerstone of DCM outcome. Optimal pharmacological and non-pharmacological decisions, mostly based on LVEF, have improved DCM prognosis drastically^16^. Major arrhythmogenic episodes, sudden death events and mortality are related to DCM with lower EF ≤ 35%^7^. The impact of stratifying the severity of systolic impairment is crucial in DCM as shown^6,7,17^, but access to the EF has several limitations. The need for expert professionals, intra- and inter-observational differences or time-consuming imaging techniques in transthoracic echocardiography or cardiac magnetic resonance, as well as inaccessible or excessive radiation imaging techniques, make it difficult to monitor the patient. The lack of a specific circulating biomarker to stratify LVEF and the limitations of cardiac imaging tests lead to the need to investigate a novel tool for this at-risk population.

Some studies have struggled to relate preserved and impaired LVEF to HF categorization^18–20^. The dysregulation of miRNAs related to DCM diagnosis and its etiology has scarcely been characterized nor categorization of the EF has not been reported in this population^14,15,21^. Some groups have worked on the reverse remodeling of LV in DCM or the signalling network^22^. To the best of our knowledge, no study has focused on LVEF in the DCM population and circulating miRNA*.*

In the present study, fourteen plasma miRNAs showed discriminative power to define DCM^SEV^ *vs*. DCM^MOD^ idiopathic patients based on their correlation to some echocardiographic variables. The idiopathic DCM^SEV^ group showed an association between LVEF with these miRNAs. In the case of DCM^MOD^ patients, the miRNAs were associated with LVESD, and LA. Remarkably, it has been suggested that pressure overload in earlier stages of DCM and elevated LV filling pressures lead to a hemodynamic imbalance that justifies the increased LA and LVESD in this cohort^23,24^.. Interestingly, some of these fourteen miRNAs have been related to HF and DCM patients with an EF < 30%, compared with healthy controls. Circulating levels of let-7a-5p were significantly upregulated in HF patients^25^. Myocardial and circulating miR-30 levels were significantly increased in DCM patients^22^. We present a novel, reliable fingerprint of circulating miRNAs to discriminate between idiopathic DCM^MOD^ and DCM^SEV^ subjects.

A multiparametric model was performed as a potential tool for the diagnosis of idiopathic DCM^SEV^. A panel comprised of a combination of three circulating miRNAs (miR-130b-3p, miR-150-5p and miR-210-3p) with four clinical variables (LBBB, LVESD, SBP and smoking habit) showed a high-yield diagnostic accuracy with an AUC of 0.96. Significantly, miR-150-5p and miR-210-3p had a positive value while miR-130b-3p had a negative value for a diagnosis of idiopathic DCM.^SEV^.

A total of 1902 genes were predicted to be targeted by these three miRNAs. Although they have been described as key hypoxia-related miRNA, our *in-silico* analysis revealed that they are more than silent players in hypoxia. Hence, these miRNAs may influence DCM^SEV^ devolvement, promoting heart injury. MiR-210-3p and miR-150-5p share targeted genes linked within the cellular proliferation, differentiation oxidative stress and apoptosis^26–29^. The miR-210 expression has been extensively studied in several cardiovascular-related diseases with controversial results^30–33^. Its overexpression reduces mitochondrial reactive oxygen species production, and promotes cardiomyocyte proliferation, cell survival and angiogenesis post-myocardial infarction^30,31^. In disagreement, Sun et al. confirmed that miR-210 induced oxidative stress and repressed mitochondrial function^34^. Our *in-silico* model showed that several mitochondrial activity-related proteins, are targets of miR-210-3p. Downregulation of these proteins may influence mitochondrial activity. Hence, our data are in agreement with previous studies that demonstrate impaired mitochondrial function in idiopathic DCM^35,36^. On the other hand, miR‐150 levels have been described as a responsive protector in ischemic heart disease^37–39^ and DCM^40^. MiR-150 protects the heart from ischemic injury by inhibiting the inflammatory response and repressing pro-apoptotic gene expression^39^. Otherwise, a different role has been defined for miR-150. Its levels were upregulated in the infarct area compared with other cardiac zones, altering the LV remodeling^37^. Furthermore, the downregulation of miR-150 targeted genes intensifies cardiac injury by deregulation of cell cycle-dependent intracellular Ca^2+^ concentration, and oxidative stress increase^29^; accompanied by an increase in reactive nitrogen disease, DNA damage and cardiomyocyte death^41^. MiR-130b-3p is negatively associated in our model. Instead of having a cytoprotective role, miR-130b-3p promotes cardiomyocytes injury^42^. Although these three miRNAs have been studied in several cardiovascular-related diseases, their role is still unknown regarding idiopathic DCM.

Using predictive tools, we report an additive value to our model, including some clinical variables such as LBBB, LVESD, SBP and smoking habit. LBBB, SBP and LVESD are a risk factor marker of HF^5,43^. Interestingly, LBBB and lower EF have widely shown a better response to non-pharmacological treatment, such as an implantable defibrillator^44–46^. Both clinical markers are related to neurohormonal activation in HF leading to severe myocardial damage^43^. Our circulating levels of miRNAs may reflect a dysregulation of the cardiomyocyte protecting processes arising from severe impairment of the LVEF in the DCM^SEV^ population.

We have described a circulating miRNA signature as a useful and non-invasive tool for LVEF classification in idiopathic DCM patients. In addition, we have defined a parametric model, a miRNA panel and four clinical parameters that categorize DCM based on LV systolic impairment with the highest diagnostic values reported to date. The bioinformatics analysis may elucidate the underlying molecular and cellular mechanisms of idiopathic DCM. Our results present a step-forward personalized therapeutic strategy and management of this entity.

There are several limitations to the current study. The study sample was small, but the patients who were included were strictly DCM subjects. Recently, it has been reported that sex differences might play a role in the prognosis of DCM^47,48^. In our case, larger sample size is needed to validate these data. Therefore, before these novel biomarkers can be routinely used in clinical practice, the data should be replicated and extended to larger populations. Secondly, data on natriuretic peptides, troponin, or reverse remodeling were not available in all patients to compare to our results. Our sample was of patients in a chronic situation, who were not hospitalized but were prospectively followed in the outpatient clinic. Future studies should include these data. The dysregulation of miRNAs related to DCM diagnosis and its etiology has scarcely been characterized and characterization of the EF has not been reported in this population. In the bioinformatic analysis, we only identified the genes targeted by miR-210-3p and miR-150-5p, since both are positively related to our logistic regression model. MiR-130b-3p was not included in the PPI analysis since it is negatively related to our logistic model. In addition, the analysis of 1902 genes did not show clear results due to a large number of interactions and data overload. Finally, we have no evidence that these circulating miRNAs are directly secreted into the extracellular space from the heart. Thus, dedicated experiments on human heart samples are necessary to verify these results and bioinformatic predictions.

## Conclusion

In conclusion, we identified a miRNAs fingerprint that is differentially expressed in the idiopathic DCM^SEV^ population. This signature arises as a potential clinical biomarker to discriminate DCM etiology and stratify its risk, based on the LVEF. The clinical usefulness of this miRNA panel as a diagnostic tool could lead to tailored treatment improving the DCM population outcome. In addition, the bioinformatics analysis results may be useful in elucidating the underlying mechanisms of idiopathic DCM.

## Methods

### Study population and design

This was a prospective cross-sectional study. Inclusion criteria were patients with clinical features of DCM^1^, a LV end-diastolic diameter larger than 56 mm and a LVEF below 50%. Exclusion criteria were genetic DCM or any cardiovascular, life-limiting systemic condition or an infectious or tumoral condition that could influence the DCM definition or miRNA results. Only patients older than 18 years old were enrolled. A total of 159 consecutive subjects were included in the study: (1) 55 idiopathic DCM patients, (2) 60 ischemic DCM and (3) 44 healthy controls who had been referred to the Cardiology Department of the University Hospital Puerta del Mar, Cádiz, Spain. The study design is shown in Fig. 1. LVEF was classified into two categories based on prior studies: ≤ 30%, severely reduced EF (DCM^SEV^) and 31–49% moderately reduced EF (DCM^MOD^)^49,50^. A transthoracic echocardiography protocol was performed as described previously^13,15^. Echocardiographic methods for the measurement of EF were the apical biplane method of disks (modified Simpson’s rule) and when this was not possible, Teicholz^51^. Detailed anthropometric, clinical and pharmacological information was obtained from each subject including family history, symptoms of HF, an electrocardiogram, a 24-h Holter electrocardiogram and, when appropriate, cardiac magnetic resonance. Our institution’s ethics committee (Comité de Ética de la Investigación de Cádiz) approved the study protocol. The study was performed in full compliance with the Helsinki II Declaration. All participants provided written informed consent.

### Blood collection

Ten millilitres of peripheral blood were collected in K2-ethylenediaminetetraacetic acid tubes (BD) after 10 h overnight fasting and were immediately centrifuged (1500 xg, 15 min, 4 °C). The blood was processed within 4 h after isolation. The upper layer containing plasma was divided into aliquots and stored at -80ºC until further analysis.

### RNA isolation

Total RNA was isolated using a miRNeasy Serum/Plasma Kit (Qiagen), according to the manufacturer’s instructions. A total of 200 µL of plasma aliquots were used to obtain enough final volume. To normalize extracellular miRNAs, a mix of synthetic *Caenorhabditis Elegans* miR-39-3p (cel-miR-39-3p) (1.6 × 10^8^ copies/µL) (Qiagen) and UniSp6 (Qiagen) was added to each sample. RNA was stored at -80 °C.

### MicroRNA real-time reverse transcriptase-polymerase chain reaction

RNA was reverse transcribed using the miRCURY LNA RT Kit (Qiagen) as previously described^15^. The reverse transcription reaction was performed under the following conditions: 60 min at 42 ºC, heat-inactivated for 5 min at 95 ºC and immediately cooled to 4 ºC. Then cDNA was stored at − 20 ºC. Quantitative real-time PCR (qPCR) was performed according to the protocol of miRCURY LNA SYBR Green PCR Kit (Qiagen). CFX Connect PCR System (BioRad) was used to perform the qPCR, at 95 °C for 2 min, followed by 40 cycles of 95 °C for 10 s, and 56 °C for 60 s, followed by melting curve analysis. qPCR amplification curves were evaluated with CFX Manager™ software (BioRad). The specificity of the amplification was corroborated by melting curve analysis.

The miRCURY LNA miRNA Serum/Plasma Focus PCR Panel (Qiagen) was used for the screening study. This panel contains 179 miRNA primer sets expressed in human serum/plasma. Then, custom 96-wells Pick-&-Mix microRNA PCR plates (Qiagen) were used to validate each miRNA candidate. To account for the variability between the plates, the interplate calibrator UniSp3 was analysed. Cqs above 35 cycles were censored at the minimum level observed for each miRNA. cel-miR-39-3p levels were stable across all samples. Relative quantification was performed using the 2 − ΔCq method, where ΔCq = CqmiRNA − Cqcel-miR-39-3p miRNA levels were log-transformed before being used in the statistical analyses. We have previously used this approach in the field of circulating non-coding RNAs^13,15^.

### miRNA-gene network analysis

The miRNAs obtained were tested using the miRDB database (http://mirdb.org/) to predict the targeted genes^52^. A database analysis to identify the biological function was performed using GO enrichment analysis (http://geneontology.org/)^53^ and the KEGG (https://www.kegg.jp/kegg/) software^54^. R software (www.r-project.org) was assessed to build up miRNA-mRNA^55^. The STRING database (http://www.string-db.org/) was used to analyse the PPI networks^56^.

### Statistical analysis

Continuous variables are shown as mean ± standard deviation. Categorical variables are expressed as frequency and percentage of patients (%). Intergroup comparisons of miRNAs levels were performed using non-parametric Mann–Whitney and Kruskal–Wallis rank tests for continuous variables. The Pearson correlation coefficient was used for correlations between echocardiographic and clinical parameters *vs.* log_2_ miRNAs. An analysis of differences between groups was performed using analysis of variance. Receiver operating characteristic (ROC) curves that characterize the diagnostic performance of candidate miRNAs and logistic regression models were plotted to determine the area under the curve (AUC) and the specificity and sensitivity of the optimal cut-offs. ROC curves were generated by plotting sensitivity against 100-specificity. Data were presented as the AUC and 95% confidence intervals. The relationships between miRNAs and LVEF status were assessed using logistic regression. Multiple logistic regression modelling was applied to perform the miRNA panel. The regression coefficients of each miRNA that was significantly associated with the outcome were applied to estimate the miRNA panel value. The Wilcoxon test and iterating combinations between our miRNA candidates, as well as echocardiographic and clinical covariates, were used to construct several models. The changes in *p*-values of their variables were evaluated by the Wald test and a likelihood ratio. The statistical software package R (www.r-project.org) was used for all analyses^55^.

## Supplementary Information

Supplementary Information

## Data Availability

The authors confirm that the data supporting the findings of this study are available within the article and its supplementary materials.

## Abbreviations

AUC: Area under the curve
AIC: Akaike Information Criterion
CI: Confidence interval
EF: Ejection fraction
DCM: Dilated cardiomyopathy
GO: Gene Ontology
HF: Heart failure
ISCU: Iron-sulphur cluster homolog
KEGG: Kyoto Encyclopedia of Genes and Genomes
LA: Left atrial dimension
LBBB: Left bundle branch block
LV: Left ventricle
LVEF: Left ventricle ejection fraction
LVESD: LV end-systolic dimension
miRNA: MicroRNA
OR: Odd ratio
PPI: Protein–protein interaction networks
qPCR: Quantitative real-time PCR
ROC: Receiver-operator characteristic
SBP: Systolic blood pressure
STRING: Search Tool for the Retrieval of Interacting Genes
