# Supplementary material for: Plasma microrna expression profile for reduced ejection fraction in dilated cardiomyopathy
Source: Sci Rep. 2021 Apr 6;11:7517. doi: 10.1038/s41598-021-87086-1 (PMC8024336; doi:10.1038/s41598-021-87086-1)
Supplement: Supplementary file 1 — Supplementary Information [file 41598_2021_87086_MOESM1_ESM.pdf]

SUPPLEMENTARY INFORMATION

TITLE: PLASMA MICRORNA EXPRESSION PROFILE FOR REDUCED EJECTION FRACTION IN DILATED CARDIOMYOPATHY

Maria Calderon-Dominguez[1*]. Thalía Belmonte[1]. Maribel Quezada-Feijoo[2.3]. Mónica Ramos[2.3]. Juan Calderon-Dominguez[1]. Oscar Campuzano[5-7]. Alipio Mangas[1.8.9]. Rocio Toro[1.9*]

[1]Biomedical Research and Innovation Institute of Cadiz (INiBICA). Research Unit. Puerta del Mar University Hospital. Cádiz. Spain

[2]Cardiology Department. Cruz Roja Hospital. Madrid. Spain

[3]Universidad Alfonso X. Madrid. Spain

[5] Cardiovascular Genetics Center. University of Girona-IDIBGI. Girona. Spain [6]Medical Science Department. School of Medicine. University of Girona. Girona. Spain

[7]Centro de Investigación Biomédica en Red. Enfermedades Cardiovasculares (CIBERCV). Spain

[8]Internal Medicine Department. Puerta del Mar University Hospital. School of Medicine. University of Cadiz. Spain

[9] Medicine Department. School of Medicine. University of Cadiz. Cádiz. Spain

Supplemental figure 1

FIGURE S1.

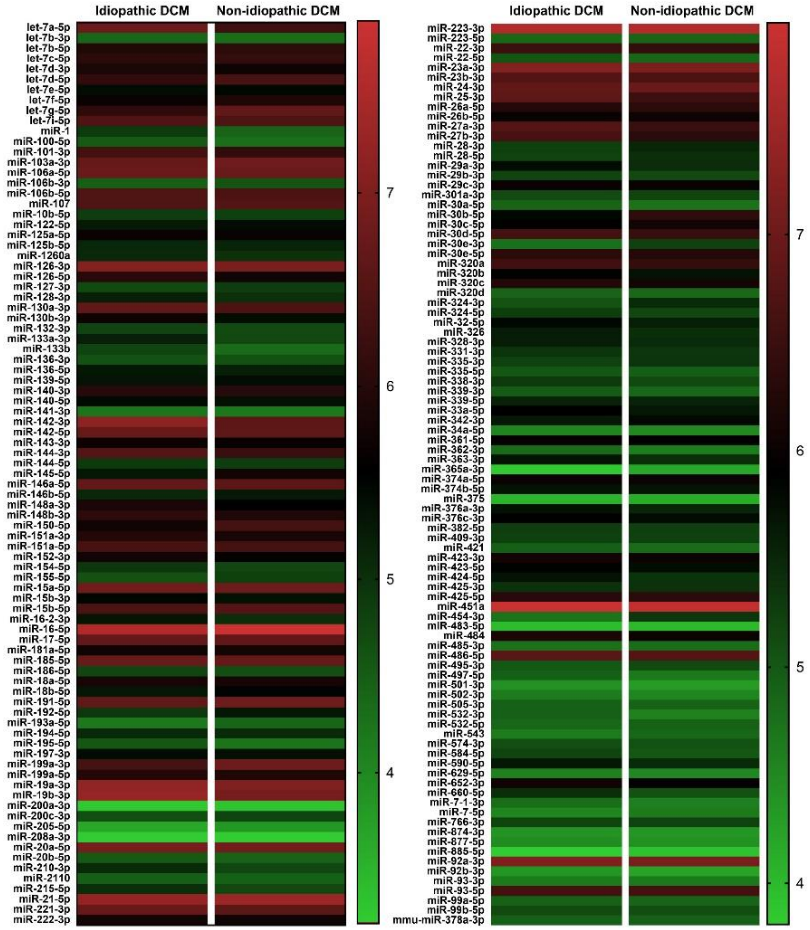

Supplemental figure 2

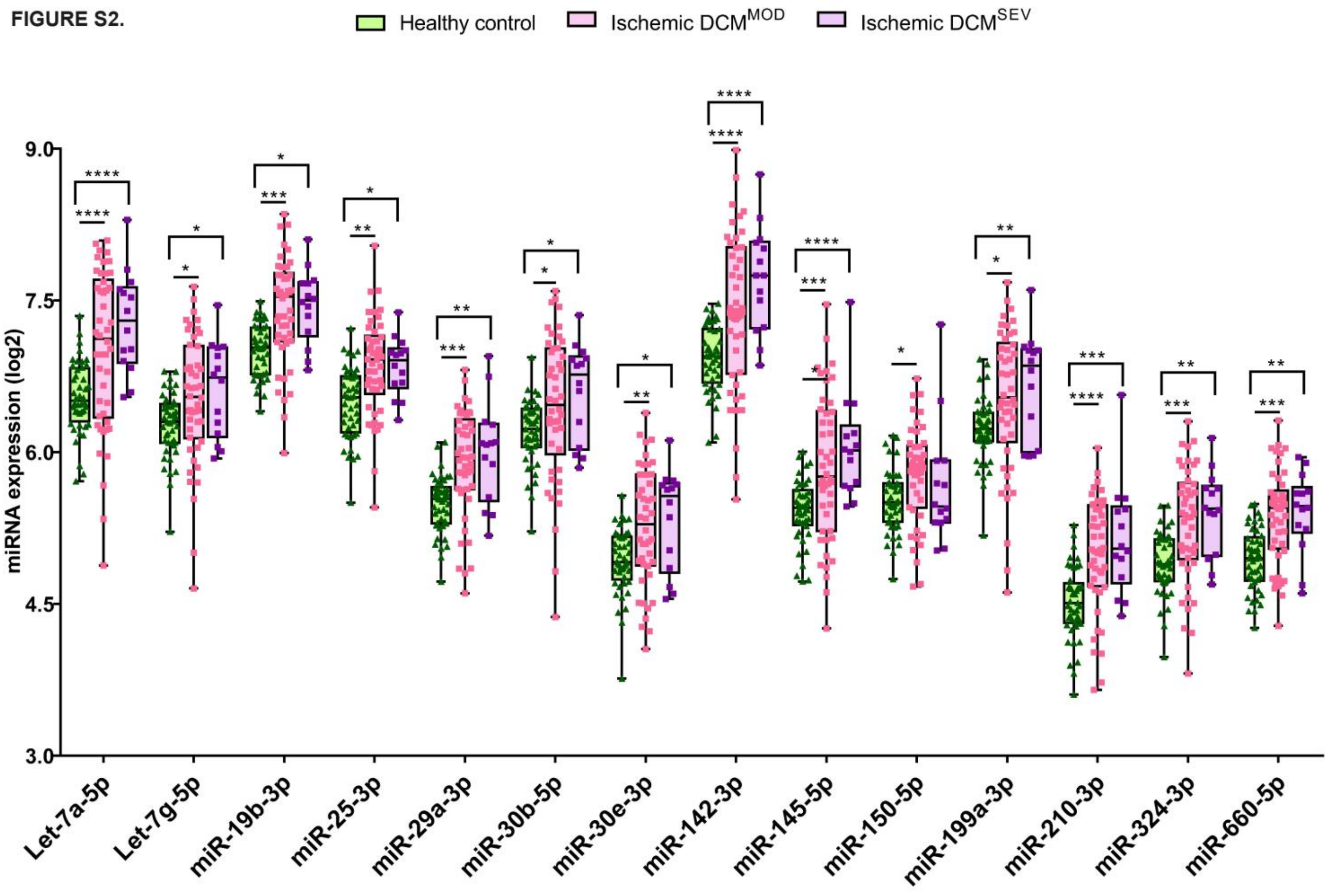

Supplemental figure 3

FIGURE S3.

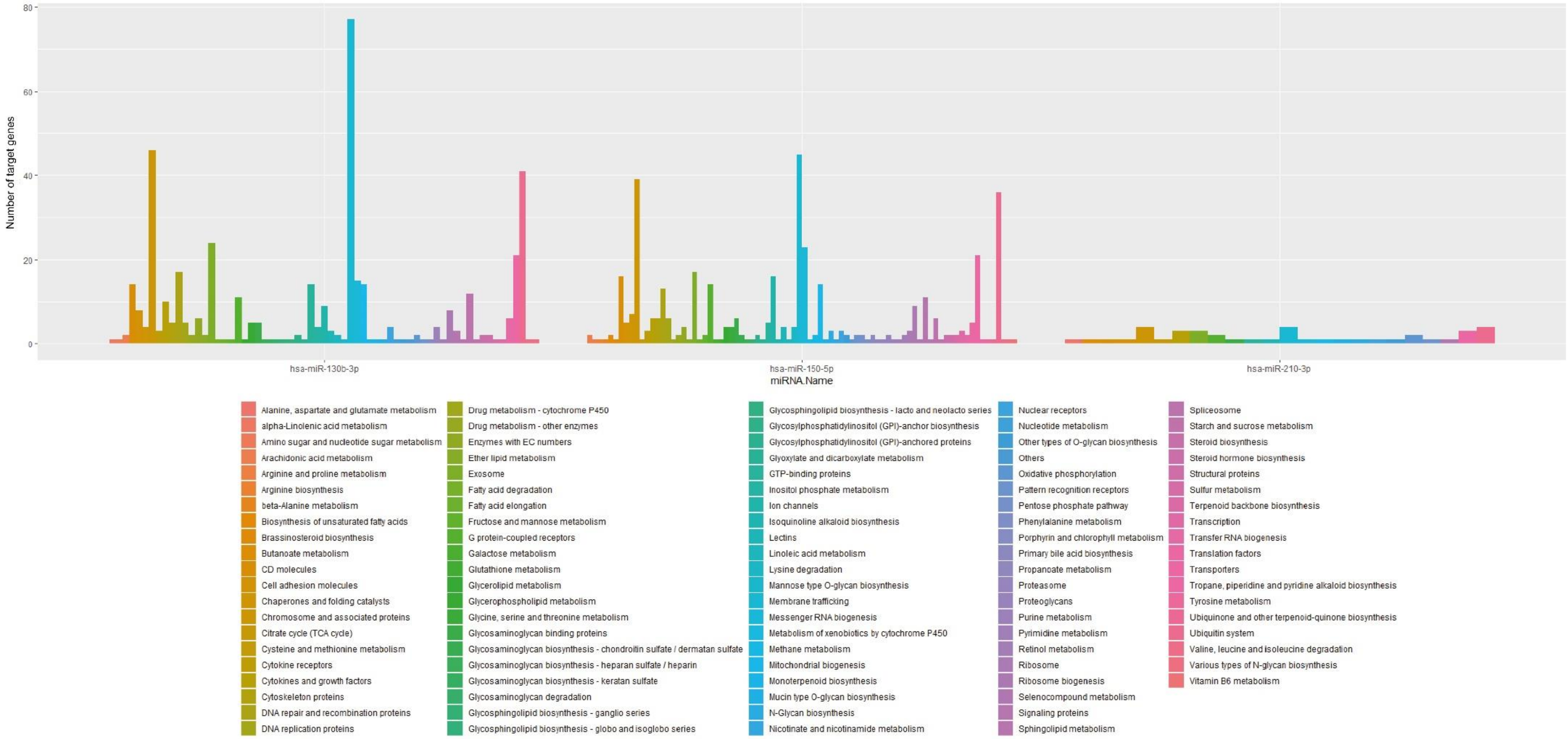

Supplemental figure 4

FIGURE S4.

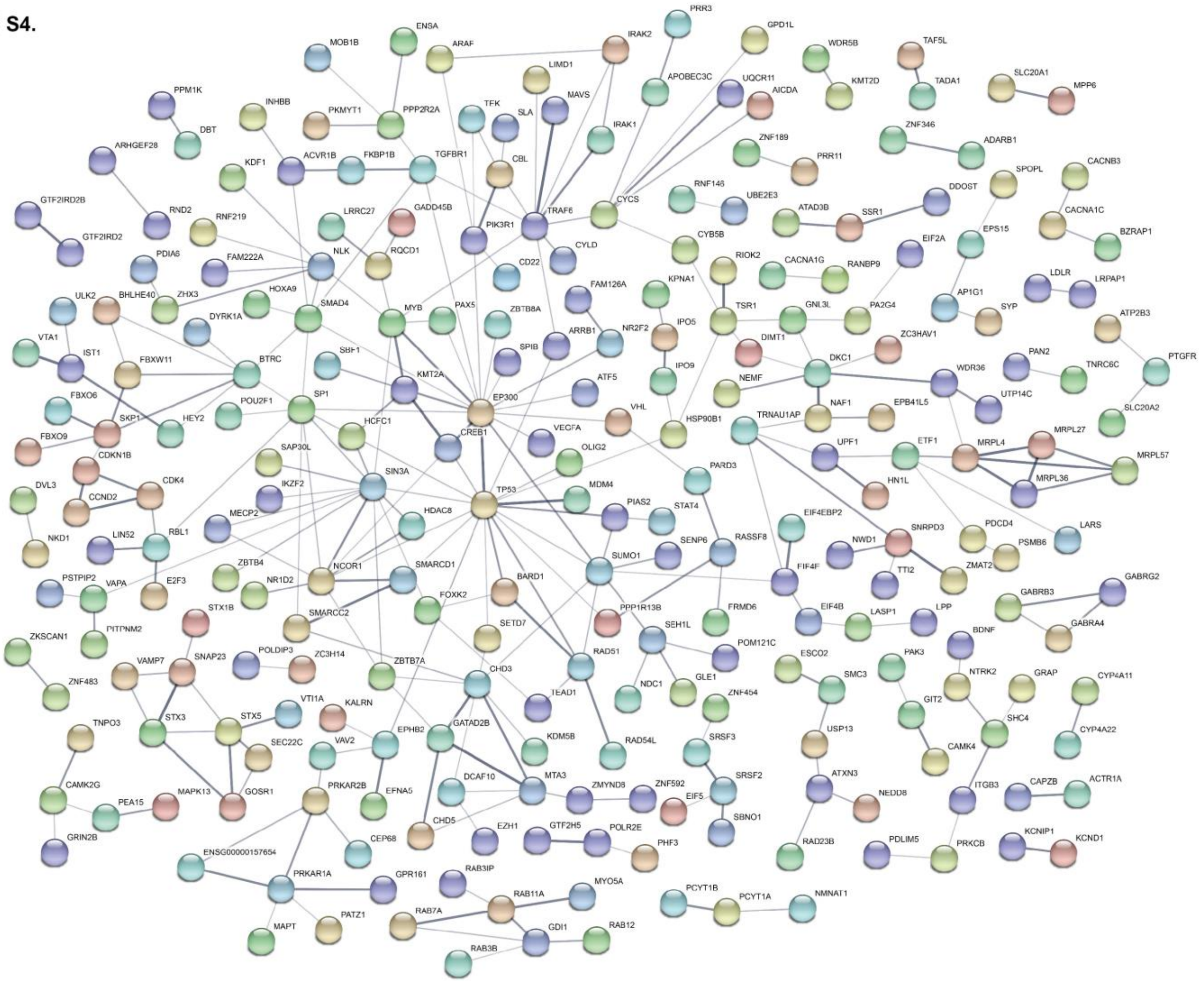

Supplemental Table 1

Supplemental Table 1. microRNAs levels (screening).			
**miRNA**	**Idopathic DCM Median (Q1-Q3)**	**Non-idiopathic DCM Median (Q1-Q3)**	***p* value**
**let-7a-5p**	6.740 (6.338 - 7.178)	6.385 (6.080 - 6.535)	0.0487
**let-7b-3p**	4.346 (4.054 - 4.660)	4.132 (4.024 - 4.679)	0.4764
**let-7b-5p**	5.845 (5.625 - 6.443)	6.205 (5.646 - 6.601)	0.4487
**let-7c-5p**	6.165 (5.670 - 6.422)	6.274 (5.498 - 6.618)	0.7783
**let-7d-3p**	5.763 (5.484 - 6.276)	5.680 (5.464 - 6.050)	0.5345
**let-7d-5p**	6.067 (5.223 - 6.780)	6.353 (6.183 - 6.655)	0.3127
**let-7e-5p**	5.477 (4.804 - 5.940)	5.485 (4.980 - 5.863)	0.848
**let-7f-5p**	5.447 (4.809 - 6.493)	5.866 (5.684 - 6.187)	0.4626
**let-7g-5p**	6.098 (5.551 - 6.807)	6.495 (6.280 - 7.120)	0.0452
**let-7i-5p**	6.432 (6.047 - 6.844)	6.381 (6.099 - 6.791)	0.9551
**miR-1**	4.834 (4.638 - 5.391)	4.473 (3.943 - 4.767)	0.018
**miR-100-5p**	4.550 (4.232 - 4.736)	4.234 (3.933 - 4.461)	0.0875
**miR-101-3p**	6.329 (5.938 - 6.659)	6.067 (5.737 - 6.478)	0.2515
**miR-103a-3p**	6.654 (6.087 - 7.344)	6.709 (6.555 - 7.221)	0.7436
**miR-106a-5p**	6.752 (6.243 - 7.257)	6.656 (6.448 - 7.069)	0.9911
**miR-106b-3p**	4.292 (3.965 - 4.910)	4.559 (4.235 - 4.990)	0.5199
**miR-106b-5p**	6.377 (5.985 - 6.954)	6.265 (6.041 - 6.765)	0.8303
**miR-107**	6.479 (5.999 - 6.924)	6.546 (6.061 - 6.859)	0.8304
**miR-10b-5p**	4.819 (4.646 - 5.067)	4.819 (4.507 - 5.046)	0.7268
**miR-122-5p**	5.300 (4.650 - 5.706)	5.400 (4.772 - 5.863)	0.3353
**miR-125a-5p**	5.714 (5.081 - 6.229)	5.552 (5.360 - 5.965)	0.973

**miR-125b-5p**	5.060	(	4.764	-	5.402	)	5.011	(	4.815	-	5.359	)	0.848
**miR-1260a**	5.164	(	4.510	-	5.619	)	5.324	(	4.060	-	5.584	)	0.955
**miR-126-3p**	7.034	(	6.590	-	7.593	)	6.823	(	6.651	-	7.360	)	0.6603
**miR-126-5p**	5.972	(	5.573	-	6.526	)	5.771	(	5.334	-	6.272	)	0.4026
**miR-127-3p**	4.336	(	3.932	-	5.495	)	4.849	(	4.368	-	5.229	)	0.4088
**miR-128-3p**	5.112	(	4.815	-	5.545	)	4.979	(	4.776	-	5.321	)	0.3591
**miR-130a-3p**	6.580	(	6.407	-	7.036	)	6.274	(	6.124	-	6.735	)	0.1608
**miR-130b-3p**	5.660	(	5.307	-	6.059	)	5.260	(	5.040	-	5.790	)	0.0138
**miR-132-3p**	4.737	(	4.412	-	5.177	)	4.694	(	4.368	-	5.102	)	0.7433
**miR-133a-3p**	5.101	(	4.887	-	5.639	)	4.620	(	4.284	-	5.156	)	0.0283
**miR-133b**	4.748	(	4.539	-	5.275	)	4.180	(	3.997	-	4.651	)	0.0361
**miR-136-3p**	4.706	(	3.651	-	5.434	)	4.635	(	3.944	-	5.150	)	0.9553
**miR-136-5p**	4.915	(	4.712	-	6.165	)	5.147	(	4.746	-	5.630	)	0.8747
**miR-139-5p**	5.132	(	4.790	-	5.966	)	5.381	(	5.070	-	5.984	)	0.7015
**miR-140-3p**	5.957	(	5.684	-	6.326	)	5.920	(	5.643	-	6.291	)	0.7267
**miR-140-5p**	5.431	(	4.951	-	5.815	)	5.244	(	5.037	-	5.645	)	0.7958
**miR-141-3p**	4.226	(	3.618	-	4.938	)	4.145	(	3.854	-	4.621	)	0.991
**miR-142-3p**	7.120	(	6.595	-	7.705	)	6.640	(	6.375	-	6.860	)	0.0345
**miR-142-5p**	6.724	(	6.277	-	7.264	)	6.482	(	6.295	-	6.854	)	0.8129
**miR-143-3p**	5.549	(	5.236	-	6.242	)	5.609	(	5.208	-	5.878	)	0.9192
**miR-144-3p**	6.463	(	6.200	-	6.841	)	6.087	(	5.716	-	6.649	)	0.1408
**miR-144-5p**	4.877	(	4.539	-	5.113	)	4.872	(	4.585	-	5.118	)	0.8835
**miR-145-5p**	5.385	(	4.988	-	5.648	)	5.775	(	5.418	-	6.098	)	0.0432
**miR-146a-5p**	6.578	(	6.297	-	7.169	)	6.540	(	6.322	-	6.965	)	0.9735
**miR-146b-5p**	5.102	(	4.705	-	5.502	)	5.214	(	4.713	-	5.685	)	0.6117
**miR-148a-3p**	5.832	(	5.444	-	6.378	)	5.527	(	5.110	-	6.002	)	0.2424
**miR-148b-3p**	6.017	(	5.791	-	6.395	)	5.914	(	5.625	-	6.226	)	0.4224

**miR-150-5p**	5.738	(5.408	-	5.906)		6.090	(	5.780	-	6.670	)	0.0427
**miR-151a-3p**	5.762	(5.623	-	6.367)		5.877	(	5.417	-	6.222	)	0.9192
**miR-151a-5p**	6.315	(5.813	-	6.996)		6.232	(	6.077	-	6.798	)	>0.9999
**miR-152-3p**	5.669	(5.446	-	6.112)		5.601	(	5.219	-	5.941	)	0.396
**miR-154-5p**	4.791	(4.465	-	5.467)		4.698	(	4.172	-	5.210	)	0.4907
**miR-155-5p**	4.586	(4.128	-	5.139)		4.684	(	4.516	-	5.287	)	0.4087
**miR-15a-5p**	6.787	(6.491	-	7.220)		6.701	(	6.502	-	7.117	)	0.7352
**miR-15b-3p**	5.456	(5.146	-	5.789)		5.339	(	5.047	-	5.625	)	0.4623
**miR-15b-5p**	6.595	(5.955	-	7.119)		6.558	(	5.950	-	6.925	)	0.9372
**miR-16-2-3p**	5.420	(5.030	-	5.510)		4.990	(	4.773	-	5.295	)	0.0281
**miR-16-5p**	7.560	(7.423	-	7.690)		7.915	(	7.610	-	8.180	)	0.0257
**miR-17-5p**	6.643	(6.215	-	7.132)		6.585	(	6.332	-	7.041	)	0.973
**miR-181a-5p**	5.674	(5.200	-	6.236)		5.823	(	5.488	-	6.139	)	0.7015
**miR-185-5p**	6.677	(6.326	-	7.102)		6.668	(	6.455	-	6.940	)	0.9461
**miR-186-5p**	4.849	(4.103	-	5.526)		4.787	(	3.897	-	5.201	)	0.7954
**miR-18a-5p**	5.812	(5.256	-	6.391)		5.716	(	5.491	-	6.114	)	>0.9999
**miR-18b-5p**	5.255	(4.813	-	5.941)		5.372	(	5.092	-	5.852	)	0.4017
**miR-191-5p**	6.668	(5.897	-	7.207)		6.663	(	6.517	-	7.180	)	0.5495
**miR-192-5p**	4.920	(4.680	-	5.180)		5.235	(	4.953	-	5.580	)	0.0398
**miR-193a-5p**	4.238	(3.728	-	4.495)		4.352	(	4.177	-	4.555	)	0.3241
**miR-194-5p**	5.011	(4.789	-	5.452)		4.998	(	4.774	-	5.484	)	0.7612
**miR-195-5p**	4.524	(4.300	-	5.031)		4.317	(	3.653	-	4.591	)	0.1226
**miR-197-3p**	5.465	(4.901	-	5.830)		5.258	(	5.107	-	5.676	)	0.9371
**miR-199a-3p**	6.280	(6.065	-	6.970)		6.735	(	6.413	-	7.220	)	0.0417
**miR-199a-5p**	5.996	(5.398	-	6.508)		5.755	(	5.603	-	6.213	)	0.9547
**miR-19a-3p**	7.201	(6.868	-	7.573)		6.946	(	6.658	-	7.356	)	0.2514
**miR-19b-3p**	7.260	(6.918	-	7.638)		6.845	(	6.680	-	7.195	)	0.0304

**miR-200a-3p**	3.138 (3.138 - 3.207)	3.138 (3.138 - 3.490)	0.9768
**miR-200c-3p**	4.720 (3.949 - 5.143)	4.737 (4.583 - 5.100)	0.744
**miR-205-5p**	3.724 (3.553 - 3.763)	3.783 (3.553 - 4.162)	0.244
**miR-208a-3p**	3.220 (3.220 - 3.220)	3.220 (3.220 - 3.220)	>0.9999
**miR-20a-5p**	6.869 (6.502 - 7.319)	6.849 (6.581 - 7.196)	0.8129
**miR-20b-5p**	4.631 (4.139 - 4.986)	4.532 (3.869 - 4.993)	0.6934
**miR-210-3p**	4.966 (4.724 - 5.470)	4.685 (4.500 - 4.983)	0.0493
**miR-2110**	4.341 (4.102 - 4.951)	4.266 (4.101 - 4.743)	>0.9999
**miR-215-5p**	5.005 (4.698 - 5.358)	4.695 (4.570 - 4.855)	0.0266
**miR-21-5p**	7.195 (6.962 - 7.682)	7.363 (6.912 - 7.923)	0.9347
**miR-221-3p**	6.588 (6.312 - 7.164)	6.498 (6.323 - 6.905)	0.6275
**miR-222-3p**	5.632 (5.469 - 6.127)	5.621 (5.380 - 6.043)	0.5802
**miR-223-3p**	7.734 (7.157 - 8.241)	7.492 (7.326 - 8.024)	0.9376
**miR-223-5p**	4.653 (4.497 - 5.151)	4.806 (4.455 - 5.147)	0.919
**miR-22-3p**	6.530 (6.221 - 6.944)	6.390 (6.074 - 6.673)	0.5196
**miR-22-5p**	5.086 (4.619 - 5.397)	4.758 (4.523 - 5.146)	0.313
**miR-23a-3p**	7.183 (6.689 - 7.615)	7.052 (6.873 - 7.446)	0.8393
**miR-23b-3p**	6.608 (6.256 - 7.164)	6.664 (6.356 - 6.955)	0.9013
**miR-24-3p**	6.803 (6.580 - 7.406)	6.902 (6.570 - 7.495)	0.8896
**miR-25-3p**	6.885 (6.548 - 7.158)	6.550 (6.375 - 6.618)	0.0495
**miR-26a-5p**	6.323 (5.775 - 6.853)	6.488 (5.646 - 7.016)	0.9103
**miR-26b-5p**	5.873 (5.583 - 6.551)	6.204 (5.172 - 6.672)	0.7433
**miR-27a-3p**	6.820 (6.245 - 7.269)	6.466 (6.134 - 6.876)	0.3021
**miR-27b-3p**	6.685 (6.134 - 7.051)	6.295 (5.892 - 6.720)	0.1824
**miR-28-3p**	5.188 (4.825 - 5.857)	5.409 (5.173 - 6.024)	0.3772
**miR-28-5p**	5.131 (4.418 - 5.999)	5.307 (5.114 - 5.820)	0.5802
**miR-29a-3p**	6.080 (5.550 - 6.170)	5.340 (5.210 - 5.748)	0.0461

**miR-29b-3p**	5.213	(	4.850	-	5.665	)	4.855	(	4.769	-	5.351	)	0.5496
**miR-29c-3p**	6.018	(	5.622	-	6.508	)	5.934	(	5.626	-	6.323	)	0.8305
**miR-301a-3p**	5.152	(	4.560	-	5.691	)	5.054	(	4.866	-	5.576	)	0.9192
**miR-30a-5p**	4.835	(	4.486	-	5.474	)	4.772	(	4.176	-	5.148	)	0.6766
**miR-30b-5p**	5.740	(	5.240	-	6.530	)	6.305	(	5.994	-	6.824	)	0.0379
**miR-30c-5p**	5.963	(	5.337	-	6.561	)	5.947	(	5.804	-	6.347	)	0.7267
**miR-30d-5p**	6.439	(	6.249	-	6.952	)	6.484	(	6.068	-	6.954	)	0.6604
**miR-30e-3p**	4.550	(	4.240	-	5.285	)	5.120	(	4.805	-	5.695	)	0.0446
**miR-30e-5p**	6.261	(	5.966	-	6.760	)	6.254	(	5.810	-	6.608	)	0.6929
**miR-320a**	6.513	(	6.201	-	6.973	)	6.445	(	5.922	-	6.763	)	0.5958
**miR-320b**	5.835	(	5.381	-	6.376	)	5.816	(	5.255	-	6.218	)	0.66
**miR-320c**	6.247	(	5.799	-	6.770	)	6.214	(	5.705	-	6.579	)	0.6275
**miR-320d**	5.151	(	3.559	-	5.656	)	5.156	(	3.981	-	5.546	)	>0.9999
**miR-324-3p**	4.950	(	4.735	-	5.500	)	5.420	(	5.045	-	5.875	)	0.0364
**miR-324-5p**	5.170	(	4.841	-	5.901	)	5.010	(	4.795	-	5.523	)	0.7613
**miR-32-5p**	5.800	(	5.553	-	6.229	)	5.457	(	5.308	-	5.925	)	0.1505
**miR-326**	5.552	(	5.206	-	6.064	)	5.254	(	5.090	-	5.694	)	0.3241
**miR-328-3p**	5.587	(	5.132	-	6.003	)	5.374	(	5.151	-	5.845	)	0.5957
**miR-331-3p**	5.380		4.842	-	5.897	)	5.176		4.943	-	5.727	)	0.9192
**miR-335-3p**	5.159		4.762	-	5.632	)	5.284		5.019	-	5.605	)	0.4904
**miR-335-5p**	5.234	(	4.238	-	5.754	)	5.054	(	4.441	-	5.462	)	0.7694
**miR-338-3p**	5.237	(	4.823	-	5.728	)	4.985	(	4.771	-	5.599	)	0.5197
**miR-339-3p**	4.979	(	4.480	-	5.424	)	4.719	(	4.548	-	5.248	)	0.6728
**miR-339-5p**	5.641	(	4.935	-	6.155	)	5.473	(	5.159	-	5.899	)	0.9908
**miR-33a-5p**	5.931	(	5.501	-	6.263	)	5.514	(	5.279	-	6.071	)	0.2422
**miR-342-3p**	5.455	(	5.283	-	6.200	)	5.628	(	5.400	-	6.150	)	0.8837
**miR-34a-5p**	4.467	(	4.140	-	4.942	)	4.399	(	4.211	-	4.671	)	0.8837

**miR-361-5p**	5.806	(	5.532	-	6.447	)	5.757	(	5.508	-	6.226	)	0.7695
**miR-362-3p**	4.802	(	4.475	-	5.079	)	4.474	(	4.306	-	5.035	)	0.261
**miR-363-3p**	5.670	(	5.480	-	5.920	)	5.345	(	5.178	-	5.718	)	0.0428
**miR-365a-3p**	3.925	(	3.456	-	4.200	)	4.058	(	3.880	-	4.510	)	0.2108
**miR-374a-5p**	6.063	(	5.463	-	6.559	)	5.964	(	5.640	-	6.542	)	0.9103
**miR-374b-5p**	5.675	(	5.168	-	6.313	)	5.634	(	5.159	-	6.216	)	0.973
**miR-375**	3.959	(	3.725	-	4.369	)	3.970	(	3.876	-	4.476	)	0.5956
**miR-376a-3p**	5.712	(	5.076	-	6.187	)	5.553	(	5.108	-	6.024	)	0.4224
**miR-376c-3p**	5.766	(	5.438	-	6.538	)	5.684	(	5.115	-	6.271	)	0.7098
**miR-382-5p**	5.117	(	4.818	-	5.710	)	5.300	(	4.735	-	5.626	)	0.8602
**miR-409-3p**	5.051	(	4.643	-	5.809	)	5.244	(	4.660	-	5.682	)	0.8479
**miR-421**	4.789	(	4.551	-	5.402	)	4.687	(	4.525	-	5.235	)	0.6439
**miR-423-3p**	6.030	(	5.601	-	6.459	)	5.882	(	5.716	-	6.452	)	0.8658
**miR-423-5p**	5.775	(	5.554	-	6.143	)	5.776	(	5.290	-	6.085	)	0.6765
**miR-424-5p**	5.822	(	5.176	-	6.101	)	5.485	(	4.807	-	5.774	)	0.1477
**miR-425-3p**	5.369	(	4.921	-	5.767	)	5.242	(	5.088	-	5.621	)	0.7959
**miR-425-5p**	6.278	(	5.857	-	6.665	)	6.198	(	6.033	-	6.599	)	0.7608
**miR-451a**	8.104	(	7.564	-	8.270	)	7.812	(	7.472	-	8.253	)	0.4762
**miR-454-3p**	4.690	(	4.170	-	5.395	)	5.315	(	4.985	-	5.513	)	0.0428
**miR-483-5p**	3.980	(	3.758	-	4.290	)	3.937	(	3.849	-	4.371	)	0.5953
**miR-484**	6.114	(	5.705	-	6.578	)	5.981	(	5.727	-	6.306	)	0.4764
**miR-485-3p**	4.480	(	4.136	-	5.411	)	4.737	(	4.346	-	5.202	)	0.733
**miR-486-5p**	6.861	(	6.449	-	7.108	)	6.664	(	6.516	-	6.966	)	0.6437
**miR-495-3p**	4.658	(	4.512	-	5.784	)	5.019	(	4.683	-	5.582	)	0.5338
**miR-497-5p**	4.835	(	4.573	-	5.238	)	4.586	(	4.472	-	4.855	)	0.1749
**miR-501-3p**	4.340	(	4.085	-	4.777	)	4.291	(	3.944	-	4.663	)	0.4625
**miR-502-3p**	4.603	(	4.366	-	5.018	)	4.447	(	4.187	-	4.788	)	0.4222

**miR-505-3p**	4.781	(	4.399	-	5.351	)	4.763	(	4.427	-	5.312	)	0.991
**miR-532-3p**	4.860	(	4.570	-	5.255	)	4.625	(	4.383	-	4.695	)	0.5957
**miR-532-5p**	4.761	(	4.535	-	5.225	)	4.821	(	4.672	-	5.110	)	0.04
**miR-543**	4.364	(	4.080	-	5.305	)	4.752	(	4.356	-	5.286	)	0.3471
**miR-574-3p**	4.904	(	4.693	-	5.508	)	4.875	(	4.726	-	5.562	)	0.9734
**miR-584-5p**	5.112	(	4.750	-	5.653	)	5.099	(	4.489	-	5.370	)	0.5648
**miR-590-5p**	5.726	(	5.460	-	5.957	)	5.320	(	5.124	-	5.842	)	0.179
**miR-629-5p**	4.529	(	4.304	-	4.883	)	4.497	(	4.122	-	4.820	)	0.5802
**miR-652-3p**	5.995	(	5.595	-	6.486	)	5.918	(	5.578	-	6.253	)	0.5646
**miR-660-5p**	5.345	(	5.210	-	5.610	)	5.060	(	4.793	-	5.403	)	0.0151
**miR-7-1-3p**	4.740	(	4.311	-	5.149	)	4.515	(	4.185	-	5.036	)	0.4906
**miR-7-5p**	4.540	(	4.057	-	5.044	)	4.735	(	4.258	-	5.086	)	0.643
**miR-766-3p**	5.165	(	4.705	-	5.728	)	4.996	(	4.913	-	5.668	)	0.8836
**miR-874-3p**	4.325	(	4.124	-	4.654	)	4.298	(	3.995	-	4.471	)	0.7609
**miR-877-5p**	4.354	(	4.098	-	4.915	)	4.369	(	4.112	-	4.692	)	0.8129
**miR-885-5p**	3.786	(	3.394	-	4.315	)	4.073	(	3.580	-	4.296	)	0.6928
**miR-92a-3p**	7.174	(	6.866	-	7.576	)	7.018	(	6.852	-	7.451	)	0.7015
**miR-92b-3p**	4.245	(	4.033	-	4.879	)	4.362	(	3.852	-	4.680	)	0.6273
**miR-93-3p**	4.529	(	4.337	-	5.085	)	4.523	(	4.383	-	4.907	)	0.9552
**miR-93-5p**	6.531	(	6.157	-	6.940	)	6.544	(	6.298	-	6.845	)	0.9189
**miR-99a-5p**	4.795	(	4.665	-	5.167	)	4.845	(	4.552	-	5.235	)	0.9193
**miR-99b-5p**	5.144	(	4.713	-	5.550	)	5.081	(	4.811	-	5.474	)	0.9551
**mmu-miR-378a-3p**	4.961	(	4.561	-	5.194	)	4.773	(	4.515	-	5.209	)	0.8656

Data presented as median (Q1-Q3). Differences between groups were analyzed using Mann-Whitney test. Abbreviations: DCM, dilated cardiomyopathy

Supplemental Table 2

Supplemental Table 2. Pearson correlation between the echocardiographic parameter and individual miRNA levels for EF subtype categorization in ischemic DCM patients.

	Ischemic DCM											
	LVEF (%)				LVESD (mm)				Left Atrium (mm)			
**microRNA**	DCM^{SEV}		DCM^{MOD}		DCM^{SEV}		DCM^{MOD}		DCM^{SEV}		DCM^{MOD}	
	r	*p*	r	*p*	r	*p*	r	*p*	r	*p*	r	*p*
**let-7a-5p**	-0.384	0.176	0.111	0.463	0.092	0.753	0.297	0.045	0.282	0.328	0.172	0.253
**let-7g-5p**	-0.204	0.485	0.138	0.359	0.076	0.795	0.248	0.096	0.499	0.069	0.124	0.410
**miR-1-3p**	0.211	0.470	0.169	0.260	0.005	0.985	0.222	0.138	0.399	0.158	-0.099	0.514
**miR-16-5p**	0.012	0.968	-0.030	0.125	-0.261	0.367	0.322	0.125	0.416	0.139	0.201	0.125
**miR-16-2-3p**	0.010	0.973	-0.047	0.259	-0.477	0.084	0.351	0.259	0.301	0.295	0.232	0.259
**miR-19b-3p**	0.027	0.927	-0.026	0.861	-0.133	0.650	0.309	0.037	0.472	0.088	0.194	0.197
**miR-25-3p**	-0.029	0.922	0.191	0.204	-0.218	0.454	0.170	0.260	0.499	0.069	0.076	0.615
**miR-29a-3p**	0.332	0.069	0.199	0.184	-0.266	0.069	0.273	0.067	0.282	0.069	0.136	0.369
**miR-30b-5p**	-0.070	0.139	0.192	0.201	0.136	0.139	0.229	0.126	0.420	0.139	0.085	0.576
**miR-30e-3p**	-0.026	0.259	0.078	0.607	0.124	0.259	0.379	0.009	0.435	0.259	0.157	0.297
**miR-130b-3p**	0.144	0.624	0.131	0.385	-0.204	0.483	0.102	0.500	0.416	0.139	-0.013	0.930
**miR-133a-3p**	-0.171	0.560	0.135	0.372	0.225	0.440	0.136	0.366	0.324	0.259	0.006	0.967
**miR-133b**	0.262	0.365	0.172	0.252	0.083	0.778	0.051	0.736	0.430	0.125	0.171	0.256
**miR-142-3p**	-0.277	0.338	0.195	0.069	-0.024	0.936	0.177	0.069	0.290	0.315	0.160	0.069
**miR-145-5p**	-0.458	0.099	0.229	0.139	0.037	0.900	0.346	0.139	0.252	0.384	0.091	0.139
**miR-150-5p**	0.086	0.769	0.000	0.158	0.060	0.838	0.306	0.158	0.320	0.264	0.185	0.158
**miR-192-5p**	0.199	0.496	0.155	0.304	-0.531	0.051	0.209	0.164	0.126	0.668	0.072	0.636
**miR-199a-3p**	0.017	0.955	0.051	0.738	0.053	0.857	0.287	0.053	0.468	0.091	0.192	0.201
**miR-210-3p**	0.214	0.463	0.014	0.924	-0.398	0.159	0.357	0.015	0.119	0.685	0.199	0.186
**miR-215-3p**	0.274	0.343	-0.009	0.952	-0.562	0.037	0.304	0.040	0.200	0.494	0.175	0.244
**miR-324-3p**	0.199	0.125	0.053	0.729	-0.126	0.125	0.238	0.112	0.533	0.125	0.139	0.356
**miR-363-3p**	0.115	0.158	-0.050	0.741	-0.404	0.158	0.329	0.025	0.492	0.158	0.220	0.142
**miR-454-3p**	-0.328	0.252	0.144	0.338	0.056	0.850	0.063	0.677	0.288	0.319	0.022	0.884
**miR-532-5p**	0.012	0.969	0.030	0.841	-0.257	0.375	0.357	0.015	0.492	0.074	0.143	0.344
**miR-629-5p**	0.215	0.460	-0.252	0.091	-0.252	0.385	0.303	0.041	0.286	0.322	0.229	0.125

**miR-660-5p**	0.090	0.760	-0.087	0.564	-0.436	0.119	0.302	0.041	0.316	0.272	0.253	0.089

Abbreviations: DCM, dilated cardiomyopathy; DCM^{MOD}, dilated cardiomyopathy with ejection fraction ≥31-49%; DCM^{SEV}, dilated cardiomyopathy with ejection fraction ≤30%; EF, ejection fraction; LVESD, LV end-systolic diameter. Coefficient significant at $p < 0.05$.

Supplemental Table 3. Pathway analysis main findings: PPI enrichment analysis based on miR-210-3p and miR-150-5p target genes

Pathway	Overlap	FDR	Genes	Database
Regulation of cellular process	605/10484	0.00027	AAK1.ACER2.ACSL4.ACTR1A.ACVR1B.ADAM22.ADARB1.ADIPOR2.ADORA2A.ADRBK2.AHI1.AICDA.AIFM2.AKAP2.ALCAM.ALOX15B.AMOTL1.ANKRD6.AP1G1.APOBEC3C.APOPT1.ARAF.ARFIP2.ARHGEF10L.ARHGEF28.ARL6IP1.ARRB1.ATAT1.ATCAY.ATF5.ATOH8.ATP6V1H.ATP8A2.ATXN3.BACH2.BARD1.BASP1.BDNF.BHLHE40.BLZF1.BORA.BSN.BTLA.BTRC.C12orf5.C2.C2CD2L.CA8.CABP4.CACNA1C.CACNA1G.CACNB3.CACNG8.CALCR.CAMK2G.CAMK4.CAMSAP1.CAMTA1.CAPZB.CAST.CBL.CBX2.CBX3.CCND2.CCR2.CD36.CD38.CD48.CD84.CDK12.CDK13.CDK14.CDK4.CDKN1B.CELF5.CERS3.CFLAR.CHD3.CHD5.CHMP4C.CHRNB2.CLEC2D.CLN8.CLSPN.CMKLR1.COMMD10.CORO2A.COX17.CPSF7.CREB1.CREB5.CSF2RA.CSRNP3.CTC1.CTH.CTSB.CXCL14.CYCS.CYLD.CYTH1.DAK.DCAF6.DCN.DENND4A.DFFA.DHX36.DHX58.DIMT1.DIO2.DKC1.DLX1.DLX6.DNAJB5.DNAJC16.DTX4.DUOX2.DUXA.DVL3.DYRK1A.E2F3.EFNA5.EGR2.EHD1.EIF2A.EIF4B.EIF4E.EIF4EBP2.EIF5.ELAVL1.ELFN2.ELK1.ENSA.ENSG00000157654.ENSG00000173575.EP300.EPB41L5.EPHA6.EPHB2.EPS15.ERCC1.EREG.ERLIN2.ERN2.ESCO2.ETF1.EZH1.FADS1.FAM101B.FAM134C.FAM168A.FAM208A.FAM212B.FBXL5.FBXO6.FBXO9.FBXW11.FCRL2.FGFRL1.FKBP14.FKBP1B.FNDC5.FOXD3.FOXK2.FOXO4.FOXP1.FRAT2.FRMD6.FSHB.FTO.FZD4.GABRA4.GABRB3.GABRG2.GADD45B.GALP.GALR2.GATAD1.GATAD2B.GCM2.GDI1.GFPT1.GGNBP2.GIGYF2.GIT2.GJD4.GLE1.GLP1R.GLUD1.GNL3L.GOLPH3L.GOSR1.GPAM.GPD1L.GPR123.GPR155.GPR161.GPR182.GPR26.GRAP.GRASP.GRHL2.GRIN2B.GRIPAP1.GTF2H5.GTF2IRD2.GTF2IRD2B.HCAR1.HCFC1.HDAC8.HEY2.HILPDA.HOOK3.HOXA2.HOXA9.HPSE2.HSP90B1.IFIT5.IGF2.IGF2BP1.IGFBPL1.IKZF2.IL13RA1.IL1A.IL7.IMPACT.ING4.INHBB.INPP5A.INPP5B.IPO5.IRAK1.IRAK2.IST1.ITGAX.ITGB3.ITIH5.ITIH6.JAM2.KALRN.KCNB1.KCNIP1.KCNJ10.KCTD20.KCTD9.KDF1.KDM5B.KIAA0319.KIAA1161.KIAA1715.KLF12.KMT2A.KMT2D.KPNA1.KREMEN1.KSR2.LAMC2.LARP1.LARS.LASP1.LDLR.LEPROT.LILRB1.LIMD1.LIN52.LRPAP1.LRRC27.LRRC38.LRRN2.LTBP2.MAMLD1.MAP4K4.MAPK13.MAPT.MARVELD3.MAVS.MBTD1.MDM4.MECP2.METAP1.MGRN1.MID1IP1.MIXL1.MLXIP.MMP14.MOB1B.MPZL1.MR1.MTA3.MTCH2.MTMR4.MUC4.MYB.MYLK3.MYO5A.MYOCD.MYPN.MZF1.NAF1.NCOR1.NEDD8.NEIL1.NEK2.NETO2.NEUROD6.NFAT5.NISCH.NKD1.NKX24.NLK.NLN.NLRP7.NME6.NMT2.NOS1.NOVA2.NPAT.NPR3.NR1D2.NR2F2.NRCAM.NRXN3.NTRK2.OLIG2.ONECUT2.OTUD3.OTUD7B.P2RX7.P2RY8.PA2G4.PAFAH1B1.PAFAH2.PAK3.PARD3.PARP16.PATZ1.PAX5.PDAP1.PDCD4.PDE1C.PDE4C.PDE7A.PDIA6.PDK4.PDLIM5.PDP1.PEA15.PERP.PI15.PIAS2.PIGR.PIK3AP1.PIK3R1.PIK3R5.PIRT.PITPNM2.PKHD1.PKMYT1.PKP4.PLAG1.PLEKHA1.PLEKHG4B.PLP2.PLXNC1.POLDIP3.POLH.POLR2E.POU2F1.POU3F2.PPARGC1A.PPFIA3.PPP1R13B.PPP2R2A.PRDM11.PRICKLE2.PRKAR1A.PRKAR2B.PRKCA.PRKCB.PRR11.PRRT2.PSAPL1.PSD3.PTCH1.PTGFR.PTGIS.PTK6.PTP4A1.PTPRN2.PURA.PVRL2.RAB11A.RAB12.RAB29.RAB3B.RAB7A.RAD23B.RAD51.RANBP9.RAP1GAP2.RASGEF1B.RASIP1.RASSF8.RBL1.RBPMS2.RC3H1.RHBDD1.RIMS2.RIOK2.RND2.RNF141.RNF146.RNF165.RORB.RQCD1.RRM2.RRP1B.RSU1.RUNX3.S1PR3.SAMD8.SAMHD1.SAP30L.SBNO1.SCIN.SCN2B.SEH1L.SEMA3A.SEMA4F.SENP6.SETD5.SETD7.SGMS1.SH3BGRL.SH3GLB1.SH3TC2.SHC4.SIN3A.SKP1.SLA.SLAIN2.SLC20A1.SLC20A2.SLC24A2.SLC30A5.SLC35B4.SLC38A3.SLC6A4.SLC8A1.SLITRK2.SMAD4.SMARCC2.SMARCD1.SMC3.SOCS7.SOGA1.SORCS3.SP1.SP5.SPIB.SPN.SPOCK1.SPOPL.SPTLC2.SREK1.SRGAP3.SRSF2.SRSF3.SSR1.SSX1.ST7L.STAR.STAT4.STK39.STOX1.STRADA.STX1B.STX3.STX5.STYXL1.SUFU.SUMO1.SUSD5.SUV39H2.SYNGR1.SYNJ2BP.SYNPO2L.SYP.TACR1.TADA1.TAF5L.TBC1D13.TBC1D14.TBC1D22B.TBX15.TEAD1.TEK.TESK2.TET3.TEX2.TGFBR1.TGIF2.THAP8.TIMM50.TLK2.TMBIM4.TMEM108.TMEM170B.TMEM64.TMX3.TNC.TNF.TNFSF8.TNPO3.TNRC6A.TNRC6B.TNRC6C.TP53.TP53INP1.TP53INP2.TPBG.TRABD2B.TRAF6.TRIL.TRIM72.TRNAU1AP.TSPAN11.TSPYL5.TTC26.UBE2J1.UBXN2B.ULK2.UNKL.UPF1.USH1C.USH2A.USP13.UST.VAMP7.VAV2.VEGFA.VEZF1.V	GO

			HL.VIPAS39.WDR36.WLS.WTAP.XBP1.ZADH2.ZBTB20.ZBTB4.ZBTB7A.ZBTB8A.ZC3H14.ZC3HAV1.ZEB1.ZFP14.ZFP30.ZFP91.ZFX.ZHX2.ZHX3.ZKSCAN1.ZMYM4.ZMYND8.ZNF131.ZNF160.ZNF182.ZNF189.ZNF229.ZNF25.ZNF317.ZNF331.ZNF335.ZNF33A.ZNF346.ZNF347.ZNF365.ZNF37A.ZNF426.ZNF430.ZNF440.ZNF445.ZNF454.ZNF460.ZNF483.ZNF486.ZNF490.ZNF500.ZNF514.ZNF555.ZNF557.ZNF568.ZNF592.ZNF625.ZNF696.ZNF700.ZNF711.ZNF773.ZNF791.ZNF793.ZNF805.ZNF84.ZNF865.ZW10.ZYG11A	
Regulation of metabolic process	402/6516	0.00032	ACER2.ACVR1B.ADAM19.ADARB1.ADIPOR2.ADORA2A.AHI1.AICDA.ANKRD6.AOC3.APOPT1.ARAF.ARL6IP1.ARRB1.ATCAY.ATF5.ATOH8.ATP6V1H.ATXN3.BACH2.BARD1.BASP1.BDNF.BHLHE40.BLZF1.BTRC.C12orf5.C2.CALCR.CAMK4.CAMTA1.CAST.CBL.CBX2.CBX3.CCND2.CCR2.CD36.CD38.CDK12.CDK13.CDK4.CDKN1B.CELF5.CERS3.CFLAR.CHD3.CHD5.CHRNB2.CLN8.CLSPN.CMKLR1.COMMD10.COX17.CPSF7.CREB1.CREB5.CSRNP3.CTC1.CTH.CYCS.CYLD.CYP4A11.DCAF6.DCN.DENND4A.DFFA.DHX36.DIMT1.DIO2.DKC1.DLX1.DLX6.DNAJB5.DUXA.DVL3.DYRK1A.E2F3.EFNA5.EGR2.EIF2A.EIF4B.EIF4E.EIF4EBP2.EIF5.ELAVL1.ELFN2.ELK1.ENSA.ENSG00000173575.EP300.EPB41L5.EPHB2.EPS15.ERCC1.EREG.ERLIN2.ERN2.ESCO2.ETF1.EZH1.FADS1.FAM168A.FAM208A.FAM212B.FBXL5.FBXW11.FKBP1B.FOXD3.FOXK2.FOXO4.FOXP1.FSHB.FZD4.GADD45B.GALR2.GATAD1.GATAD2B.GCM2.GFPT1.GGNBP2.GIGYF2.GLE1.GNL3L.GPAM.GPD1L.GRHL2.GRIN2B.GTF2H5.GTF2IRD2.GTF2IRD2B.HCFC1.HDAC8.HEY2.HOXA2.HOXA9.HSP90B1.IGF2.IGF2BP1.IKZF2.IL1A.IL7.IMPACT.ING4.INHBB.INPP5B.IPO5.IRAK1.IRAK2.IST1.ITGB3.ITIH5.ITIH6.KCTD20.KDM5B.KLF12.KMT2A.KMT2D.KPNA1.KSR2.LACTB.LARP1.LARS.LDLR.LILRB1.LIMD1.LRPAP1.MAMLD1.MAP4K4.MAPK13.MAPT.MARVELD3.MAVS.MBTD1.MDM4.MECP2.METAP1.MID1IP1.MIXL1.MLXIP.MMP14.MOB1B.MTA3.MTMR4.MYB.MYOCD.MZF1.NAF1.NCOR1.NEDD8.NEIL1.NEK2.NEUROD6.NFAT5.NKD1.NKX24.NLK.NLN.NLRP7.NOS1.NOVA2.NPAT.NPR3.NR1D2.NR2F2.NTRK2.OLIG2.ONECUT2.OPA3.OTUD7B.P2RX7.PA2G4.PAFAH1B1.PAK3.PAN2.PARD3.PARP16.PATZ1.PAX5.PDCD4.PDK4.PDP1.PEA15.PERP.PI15.PIAS2.PIK3R1.PIK3R5.PKHD1.PKMYT1.PLAG1.POLDIP3.POLH.POLR2E.POU2F1.POU3F2.PPARGC1A.PPP2R2A.PRDM11.PRKAR1A.PRKAR2B.PRKCA.PRKCB.PSAPL1.PTCH1.PTGFR.PTGIS.PTK6.PURA.RAB12.RAB29.RAB7A.RAD23B.RAD51.RANBP9.RASIP1.RBL1.RBPMS2.RC3H1.RHBDD1.RIMS2.RIOK2.RNF141.RNF165.RORB.RQCD1.RRM2.RRP1B.RUNX3.SAMD7.SAMD8.SAP30L.SBNO1.SEMA3A.SETD5.SETD7.SH3GLB1.SHC4.SIN3A.SLC35B4.SLC38A3.SLC6A4.SMAD4.SMARCC2.SMARCD1.SMC3.SOCS7.SOGA1.SP1.SP5.SPIB.SPN.SPOCK1.SPOPL.SPTLC2.SREK1.SRSF2.SRSF3.SSX1.STAR.STAT4.STK39.STOX1.STRADA.STX1B.STX5.STYXL1.SUFU.SUMO1.SUV39H2.SYNJ2BP.TADA1.TAF5L.TBC1D14.TBX15.TEAD1.TEK.TET3.TGFBR1.TGIF2.THAP8.TLK2.TNC.TNF.TNFSF8.TNRC6A.TNRC6B.TNRC6C.TP53.TP53INP1.TP53INP2.TRABD2B.TRAF6.TRNAU1AP.TSPYL5.UBE2J1.UBXN2B.UNKL.UPF1.USP13.VAV2.VEGFA.VEZF1.VHL.VIPAS39.WTAP.XBP1.ZBTB20.ZBTB4.ZBTB7A.ZBTB8A.ZC3H14.ZC3HAV1.ZEB1.ZFP14.ZFP30.ZFP91.ZFX.ZHX2.ZHX3.ZKSCAN1.ZMYM4.ZMYND8.ZNF131.ZNF160.ZNF182.ZNF189.ZNF229.ZNF25.ZNF317.ZNF331.ZNF335.ZNF33A.ZNF347.ZNF365.ZNF37A.ZNF426.ZNF430.ZNF440.ZNF445.ZNF454.ZNF460.ZNF483.ZNF486.ZNF490.ZNF500.ZNF514.ZNF555.ZNF557.ZNF568.ZNF592.ZNF625.ZNF696.ZNF700.ZNF711.ZNF773.ZNF791.ZNF793.ZNF805.ZNF84.ZNF865.ZYG11A	GO
Regulation of biosynthetic process	278/4337	0.0014	ACER2.ACVR1B.ADIPOR2.ADORA2A.AHI1.AICDA.ARRB1.ATF5.ATOH8.ATXN3.BACH2.BASP1.BHLHE40.BLZF1.BTRC.C12orf5.CAMK4.CAMTA1.CBL.CBX2.CBX3.CCR2.CD36.CD38.CDK12.CDK13.CDK4.CDKN1B.CERS3.CFLAR.CHD3.CHD5.CMKLR1.COMMD10.CREB1.CREB5.CSRNP3.CTC1.CTH.CYLD.DCAF6.DCN.DENND4A.DHX36.DIO2.DKC1.DLX1.DLX6.DNAJB5.DUXA.DVL3.E2F3.EGR2.EIF2A.EIF4B.EIF4E.EIF4EBP2.EIF5.ELAVL1.ELK1.ENSG00000173575.EP300.EREG.ERLIN2.ERN2.ESCO2.ETF1.EZH1.FADS1.FAM208A.FBXW11.FOXD3.FOXK2.FOXO4.FOXP1.FSHB.FZD4.GALR2.GATAD1.GATAD2B.GCM2.GIGYF2.GLE1.GNL3L.GPAM.GRHL2.GTF2H5.GTF2IRD2.GTF2IRD2B.HCFC1.HDAC8.HEY2.HOXA2.HOXA9.IGF2.IGF2BP1.IKZF2.IL1A.IMPACT.ING4.INHBB.IRAK1.IRAK2.ITGB3.KDM5B.KLF12.KMT2A.KMT2D.LARP1.LDLR.LILRB1.LIMD1.MAMLD1.MAPK13.MAVS.MBTD1.MDM4.MECP2.METAP1.MID1IP1.MIXL1.MLXIP.MTA3.MYB.MYOCD.M	

			ZF1.NAF1.NCOR1.NEDD8.NEK2.NEUROD6.NFAT5.NK24.NLK.NLN.NOS1.NPAT.NR1D2.NR2F2.OLIG2.ONECUT2.OTUD7B.P2RX7.PA2G4.PAK3.PATZ1.PAX5.PDCD4.PDK4.PDP1.PIAS2.PIK3R1.PKHD1.PLAG1.POLDIP3.POU2F1.POU3F2.PPARGC1A.PRDM11.PRKAR1A.PRKCB.PTCH1.PTGIS.PURA.RBL1.RBPMS2.RC3H1.RNF141.RNF165.RORB.RQCD1.RRM2.RRP1B.RUNX3.SAMD8.SAP30L.SBNO1.SETD5.SETD7.SIN3A.SLC35B4.SLC38A3.SMAD4.SMARCC2.SMARCD1.SMC3.SOGA1.SP1.SP5.SPIB.SPN.SRSF2.SSX1.STAR.STAT4.STOX1.SUFU.SUMO1.SUV39H2.TADA1.TAF5L.TBX15.TEAD1.TET3.TGFBR1.TGIF2.THAP8.TNF.TNFSF8.TNRC6A.TNRC6B.TNRC6C.TP53.TP53INP1.TP53INP2.TRAF6.TRNAU1AP.UBE2J1.UNKL.UPF1.USP13.VEGFA.VEZF1.VHL.VIPAS39.XBP1.ZBTB20.ZBTB4.ZBTB7A.ZBTB8A.ZEB1.ZFP14.ZFP30.ZFP91.ZFX.ZHX2.ZHX3.ZKSCAN1.ZMYM4.ZMYND8.ZNF131.ZNF160.ZNF182.ZNF189.ZNF229.ZNF25.ZNF317.ZNF331.ZNF335.ZNF33A.ZNF347.ZNF365.ZNF37A.ZNF426.ZNF430.ZNF440.ZNF445.ZNF454.ZNF460.ZNF483.ZNF486.ZNF490.ZNF500.ZNF514.ZNF555.ZNF557.ZNF568.ZNF592.ZNF625.ZNF696.ZNF700.ZNF711.ZNF773.ZNF791.ZNF793.ZNF805.ZNF84.ZNF865	
Regulation of RNA metabolic process	253/3890	0.0014	ACVR1B.ADORA2A.AHI1.AICDA.ARRB1.ATF5.ATOH8.ATXN3.BACH2.BARD1.BASP1.BHLHE40.BLZF1.BTRC.CAMK4.CAMTA1.CBL.CBX2.CBX3.CD36.CD38.CDK12.CDK13.CDKN1B.CELF5.CERS3.CFLAR.CHD3.CHD5.CMKLR1.COMMD10.CPSF7.CREB1.CREB5.CSRNP3.CTH.CYLD.DCAF6.DCN.DENND4A.DHX36.DIMT1.DKC1.DLX1.DLX6.DNAJB5.DUXA.DVL3.DYRK1A.E2F3.EGR2.EIF2A.ELAVL1.ELK1.ENSG00000173575.EP300.EREG.ERLIN2.ERN2.EZH1.FADS1.FAM208A.FBXW11.FOXD3.FOXK2.FOXO4.FOXP1.FSHB.FZD4.GALR2.GATAD1.GATAD2B.GCM2.GIGYF2.GRHL2.GTF2H5.GTF2IRD2.GTF2IRD2B.HCFC1.HDAC8.HEY2.HOXA2.HOXA9.IGF2.IGF2BP1.IKZF2.IL1A.IMPACT.ING4.IRAK1.IRAK2.KDM5B.KLF12.KMT2A.KMT2D.LARP1.LILRB1.LIMD1.MAMLD1.MAPK13.MAVS.MBTD1.MDM4.MECP2.MIXL1.MLXIP.MTA3.MYB.MYOCD.MZF1.NAF1.NCOR1.NEDD8.NEUROD6.NFAT5.NKX24.NLK.NOS1.NOVA2.NPAT.NR1D2.NR2F2.OLIG2.ONECUT2.OTUD7B.PA2G4.PATZ1.PAX5.PDCD4.PIAS2.PIK3R1.PKHD1.PLAG1.POU2F1.POU3F2.PPARGC1A.PRDM11.PRKAR1A.PRKCA.PRKCB.PTCH1.PTGIS.PURA.RBL1.RBPMS2.RC3H1.RIOK2.RNF141.RNF165.RORB.RQCD1.RRM2.RRP1B.RUNX3.SAP30L.SBNO1.SETD5.SETD7.SIN3A.SLC38A3.SMAD4.SMARCC2.SMARCD1.SP1.SP5.SPIB.SREK1.SRSF2.SRSF3.SSX1.STAT4.STOX1.SUFU.SUMO1.SUV39H2.TADA1.TAF5L.TBX15.TEAD1.TET3.TGFBR1.TGIF2.THAP8.TNF.TNFSF8.TNRC6B.TNRC6C.TP53.TP53INP1.TP53INP2.TRAF6.UNKL.UPF1.USP13.VEGFA.VEZF1.VHL.VIPAS39.WTAP.XBP1.ZBTB20.ZBTB4.ZBTB7A.ZBTB8A.ZC3H14.ZC3HAV1.ZEB1.ZFP14.ZFP30.ZFP91.ZFX.ZHX2.ZHX3.ZKSCAN1.ZMYM4.ZMYND8.ZNF131.ZNF160.ZNF182.ZNF189.ZNF229.ZNF25.ZNF317.ZNF331.ZNF335.ZNF33A.ZNF347.ZNF37A.ZNF426.ZNF430.ZNF440.ZNF445.ZNF454.ZNF460.ZNF483.ZNF486.ZNF490.ZNF500.ZNF514.ZNF555.ZNF557.ZNF568.ZNF592.ZNF625.ZNF696.ZNF700.ZNF711.ZNF773.ZNF791.ZNF793.ZNF805.ZNF84.ZNF865	GO
Regulation of transcription. DNA-templated	229/3661	0.0186	ACVR1B.ADORA2A.AHI1.AICDA.ARRB1.ATF5.ATOH8.ATXN3.BACH2.BASP1.BHLHE40.BLZF1.BTRC.CAMK4.CAMTA1.CBL.CBX2.CBX3.CD36.CD38.CDK12.CDK13.CDKN1B.CERS3.CFLAR.CHD3.CHD5.CMKLR1.COMMD10.CREB1.CREB5.CSRNP3.CTH.CYLD.DCAF6.DCN.DENND4A.DHX36.DLX1.DLX6.DNAJB5.DUXA.DVL3.E2F3.EGR2.EIF2A.ELK1.ENSG00000173575.EP300.EREG.ERLIN2.ERN2.EZH1.FADS1.FAM208A.FBXW11.FOXD3.FOXK2.FOXO4.FOXP1.FSHB.FZD4.GALR2.GATAD1.GATAD2B.GCM2.GRHL2.GTF2H5.GTF2IRD2.GTF2IRD2B.HCFC1.HDAC8.HEY2.HOXA2.HOXA9.IGF2.IKZF2.IL1A.IMPACT.ING4.IRAK1.IRAK2.KDM5B.KLF12.KMT2A.KMT2D.LILRB1.LIMD1.MAMLD1.MAPK13.MAVS.MBTD1.MDM4.MECP2.MIXL1.MLXIP.MTA3.MYB.MYOCD.MZF1.NCOR1.NEDD8.NEUROD6.NFAT5.NKX24.NLK.NOS1.NPAT.NR1D2.NR2F2.OLIG2.ONECUT2.OTUD7B.PA2G4.PATZ1.PAX5.PDCD4.PIAS2.PIK3R1.PKHD1.PLAG1.POU2F1.POU3F2.PPARGC1A.PRDM11.PRKAR1A.PRKCB.PTCH1.PTGIS.PURA.RBL1.RBPMS2.RNF141.RNF165.RORB.RQCD1.RRM2.RRP1B.RUNX3.SAP30L.SBNO1.SETD5.SETD7.SIN3A.SLC38A3.SMAD4.SMARCC2.SMARCD1.SP1.SP5.SPIB.SSX1.STAT4.STOX1.SUFU.SUMO1.SUV39H2.TADA1.TAF5L.TBX15.TEAD1.TET3.TGFBR1.TGIF2.THAP8.TNF.TNFSF8.TP53.TP53INP1.TP53INP2.TRAF6.UNKL.USP13.VEGFA.VEZF1.VHL.VIPAS39.XBP1.ZBTB20.ZBTB4.ZBTB7A.ZBTB8A.ZEB1.ZFP14.ZFP30.ZFP9	GO

			1.ZFX.ZHX2.ZHX3.ZKSCAN1.ZMYM4.ZMYND8.ZNF131.ZNF160.ZNF182.ZNF189.ZNF229.ZNF25.ZNF317.ZNF331.ZNF335.ZNF33A.ZNF347.ZNF37A.ZNF426.ZNF430.ZNF440.ZNF445.ZNF454.ZNF460.ZNF483.ZNF486.ZNF490.ZNF500.ZNF514.ZNF555.ZNF557.ZNF568.ZNF592.ZNF625.ZNF696.ZNF700.ZNF711.ZNF773.ZNF791.ZNF793.ZNF805.ZNF84.ZNF865	
Positive regulation of RNA metabolic process	113/1596	0.0193	ACVR1B.AHI1.AICDA.ARRB1.ATF5.ATOH8.BTRC.CAMK4.CAMTA1.CD38.CDK12.CDK13.CREB1.CREB5.CSRNP3.DCAF6.DCN.DHX36.DIMT1.DLX1.DVL3.DYRK1A.E2F3.EGR2.EIF2A.ELK1.EP300.ERLIN2.EZH1.FBXW11.FOXD3.FOXK2.FOXO4.FSHB.FZD4.GALR2.GCM2.GIGYF2.GRHL2.HCFC1.HEY2.HOXA2.IGF2.IKZF2.IL1A.ING4.IRAK1.KMT2A.KMT2D.LILRB1.MAVS.MECP2.MIXL1.MLXIP.MYB.MYOCD.MZF1.NEUROD6.NFAT5.NOS1.NPAT.NR1D2.NR2F2.ONECUT2.PATZ1.PAX5.PIAS2.PIK3R1.PLAG1.POU2F1.POU3F2.PPARGC1A.PRKCB.PTCH1.RBL1.RC3H1.RIOK2.RORB.RQCD1.RRP1B.RUNX3.SETD7.SIN3A.SLC38A3.SMAD4.SMARCC2.SMARCD1.SP1.SPIB.STAT4.TADA1.TAF5L.TEAD1.TET3.TGFBR1.TNF.TNFSF8.TNRC6B.TNRC6C.TP53.TP53INP1.TP53INP2.TRAF6.UPF1.VEGFA.VEZF1.VHL.XBP1.ZC3HAV1.ZEB1.ZFX.ZNF131.ZNF711	GO
Alternative splicing	598/10223	$2.19x10^{-06}$	AAK1.ABCB9.ABHD12.ACER2.ACSL4.ACVR1B.ADAM19.ADAM22.ADARB1.AGAP1.AHI1.AICDA.AIFM2.AIFM3.AKIRIN1.AKR1D1.ALCAM.ALDH5A1.ALOX15B.ALPK1.ALS2CR12.AMN1.AMOTL1.ANKRD12.ANKRD6.AOC3.AP1G1.ARAF.ARFIP2.ARHGEF10L.ARHGEF28.ARL6IP1.ARMC1.ARRB1.ARSA.ASTN2.ATAD3B.ATAT1.ATCAY.ATOH8.ATP2B3.ATP6V1H.ATP8A2.ATXN3.B3GNT6.BARD1.BASP1.BDNF.BEST3.BLZF1.BORA.BSDC1.BTLA.BTRC.BZRAP1.C19orf12.C1orf177.C1orf198.C2.C2CD2L.C5orf38.C8orf59.CABP4.CACNA1C.CACNA1G.CACNB3.CALCR.CALML4.CAMK2G.CAMSAP1.CAMTA1.CAPZB.CAST.CBX2.CCDC142.CCDC25.CCND2.CCR2.CD22.CD36.CD38.CD48.CD84.CDK12.CDK13.CDK14.CDK4.CELF5.CEP68.CERS5.CFLAR.CHD3.CHKA.CLEC2D.CLIC5.CLSPN.CLVS1.CMKLR1.CNTLN.CNTNAP2.CORO2B.CPD.CPSF7.CREB1.CREB5.CROT.CSF2RA.CSRNP3.CTC1.CTH.CYLD.CYP20A1.CYP4A11.CYP4A22.CYP4X1.CYTH1.DAK.DCAF10.DCAF5.DCAF6.DCN.DDOST.DENND4A.DENND5B.DFFA.DHX36.DKC1.DLX1.DLX6.DNAJB5.DNAJC16.DSG1.DTX4.DUSP13.DVL3.DYRK1A.DZANK1.E2F3.EGR2.EIF2A.EIF4B.ELAVL1.ELK1.EMC1.ENSG00000157654.ENSG00000173575.ENTPD1.EPB41L5.EPHA6.EPHB2.EPS15.ERCC1.ERLIN2.ESCO2.ETF1.EZH1.FADS1.FADS6.FAM102B.FAM126A.FAM133B.FAM134C.FAM168A.FAM168B.FAM208A.FAM227A.FAM32A.FAM63B.FAM73A.FAM73B.FBXL5.FBXO9.FBXW11.FCRL2.FCRL5.FKBP1B.FNDC5.FOPNL.FOXK2.FOXO4.FOXP1.FREM2.FRMD6.FTO.GABRB3.GABRG2.GALNT1.GALP.GDPD1.GFPT1.GGNBP2.GGT6.GIGYF2.GIT2.GK5.GLB1L.GLE1.GLT1D1.GLUD1.GLYCTK.GOLGA7.GOLPH3L.GOSR1.GPATCH2L.GPATCH8.GPR107.GPR123.GPR161.GRAMD1B.GRASP.GREB1.GRHL2.GRIPAP1.GSG1L.GTF2IRD2.GTF2IRD2B.HCFC1.HDAC8.HEATR3.HN1L.HNRNPH3.HPSE2.ICA1L.IFIT5.IGF2.IGF2BP1.IKZF2.IL13RA1.IL7.IMPACT.ING4.INPP5B.IPO5.IRAK1.ISCU.IST1.ITGB3.ITIH5.JAM2.KALRN.KANK4.KCND1.KCNJ3.KCTD20.KCTD3.KDM5B.KIAA0319.KIAA1715.KIF1A.KLF12.KMT2A.KMT2D.KREMEN1.KSR2.LACTB.LAMC2.LARP1.LARP7.LARS.LASP1.LDLR.LILRB1.LRRC27.LRRC74B.MAMDC2.MAMLD1.MAP4K4.MAPK13.MAPT.MARC2.MARCH6.MAVS.MBTD1.MDGA1.MDM4.MECP2.METTL2A.MGRN1.MGST2.MIXL1.MLC1.MLXIP.MMP16.MOB1B.MPZL1.MR1.MRPL4.MTA3.MTUS2.MUC4.MYB.MYLK3.MYO5A.MYOCD.MYOM3.MYPN.MZF1.NAF1.NBPF3.NCAM2.NCOR1.NDC1.NEK2.NEMF.NETO2.NFASC.NFAT5.NIPAL3.NISCH.NLRP7.NME6.NMNAT2.NOS1.NPR3.NR2F2.NRCAM.NRXN3.NSL1.NTRK2.NUBPL.NWD1.OSBPL9.OTUD7B.P2RX7.PA2G4.PAFAH1B1.PAK3.PAN2.PANK1.PARD3.PARD3B.PARP11.PARP16.PATZ1.PAX5.PCYOX1.PCYT1B.PDCD4.PDDC1.PDE1C.PDE4C.PDE7A.PDIA6.PDLIM5.PDP1.PEA15.PGPEP1.PHF3.PIAS2.PIK3AP1.PIK3R1.PIK3R5.PITPNM2.PKHD1.PKMYT1.PKP4.PLAG1.PLEKHA1.PLEKHM3.PLOD1.PLP2.PLXDC1.PNPO.POF1B.POLDIP3.POLH.POM121.POM121C.POU2F1.PPARGC1A.PPFIA3.PPM1K.PPP1R3A.PPP2R2A.PRDM11.PRKAR1A.PRKCB.PRR3.PRRT2.PSD3.PSTPIP2.PTCH1.PTGFR.PTK6.PTPRN2.PVRL2.PXMP4.PYCRL.R3HCC1L.RAB11A.RAB29.RAB3IP.RAD23B.RANBP9.RAP1GAP2.RASGEF1B.RASSF8.RBL1.RBM48.RBPMS2.RC3H1.RHBDD1.RIMS2.RINL.RIOK2.RNF135.RNF141.RNF146.RNF165.RPRD2.RQCD1.RRM2.RRP1B.RSU1.RUNDC3B.RUNX3.SAMD14.SAMD8.SAMHD1.SAP30L.SAYSD1.SBF1.SBNO1.SCARA3.SCD5.SCIN.SCRN3.SD	KW

			HD.SEC22C.SEH1L.SEMA4F.SENP6.SETD5.SGMS1.SH3GLB1.SH3TC2.SHC4.SIGLEC10.SIRPB2.SKP1.SLA.SLC12A5.SLC16A14.SLC1A2.SLC22A2.SLC24A2.SLC25A26.SLC25A30.SLC30A5.SLC35A2.SLC35B4.SLC35D1.SLC35E1.SLC39A14.SLC43A2.SLC4A4.SLC6A4.SLC8A1.SLITRK2.SMARCC2.SMARCD1.SMC2.SNAP23.SNRPD3.SNX7.SOCS7.SOGA1.SP1.SPIB.SPTLC3.SREK1.SRGAP3.SRSF2.SRSF3.SSR1.ST6GAL1.ST6GAL2.ST7L.STK39.STOX1.STRADA.STX1B.STX3.STX5.STYXL1.SUFU.SUMO1.SUV39H2.SV2B.SYNGR1.SYNPO2L.SYP.SZRD1.TACR1.TAF5L.TBC1D13.TBC1D14.TBX15.TDRP.TEAD1.TECPR1.TEK.TESK2.TET3.TEX2.TGFBR1.TGIF2.TIMM50.TLK2.TMC7.TMED4.TMEM108.TMEM116.TMEM168.TMEM245.TMEM44.TMEM64.TMEM87A.TMPPE.TMPRSS11A.TMX3.TNC.TNPO3.TNRC6A.TNRC6B.TNRC6C.TNS1.TP53.TP53INP1.TRIM47.TRIM66.TRIM72.TRNAU1AP.TSPEAR.TTC13.TTC26.TUFT1.UBE3B.UNKL.UPF1.USB1.USH1C.USH2A.USP13.USP31.USP49.VAMP7.VAV2.VEGFA.VHL.VIPAS39.VPS53.VTA1.VTI1A.WLS.WTAP.XBP1.XPNPEP3.XXYLT1.ZADH2.ZBTB20.ZBTB8A.ZC3H14.ZC3HAV1.ZCCHC3.ZEB1.ZFP91.ZFR2.ZFX.ZHX3.ZMYM4.ZMYND8.ZNF131.ZNF160.ZNF182.ZNF189.ZNF229.ZNF25.ZNF317.ZNF335.ZNF33A.ZNF346.ZNF347.ZNF365.ZNF460.ZNF483.ZNF500.ZNF555.ZNF557.ZNF568.ZNF625.ZNF711.ZNF773.ZNF791.ZNF793.ZNF805.ZW10.ZYG11A	
Response to oxygen levels	33/321	0.0202	C12orf5.CACNB3.CBL.CD38.CDK4.CDKN1B.CFLAR.CHRNB2.CREB1.EP300.HILPDA.HSP90B1.IRAK1.LIMD1.MDM4.MECP2.MMP14.MYB.MYOCD.NOS1.PLOD1.PPARGC1A.PTGIS.SLC6A4.SLC8A1.SMAD4.STOX1.SUV39H2.TEK.TMEM199.TP53.VEGFA.VHL	GO
Nitrogen compound metabolic process	469/8349	0.0277	AAK1.ABHD12.ACER2.ACSL4.ACVR1B.ADAM19.ADAM22.ADARB1.ADORA2A.ADPRH.ADRBK2.AICDA.ALDH5A1.ALPK1.AOC3.APOBEC3C.ARAF.ARRB1.ARSA.ATAT1.ATF5.ATOH8.ATXN3.B3GALT6.B3GNT6.B4GALT5.BACH2.BARD1.BHLHE40.BTRC.C2.CAMK2G.CAMK4.CAMTA1.CBL.CBX2.CBX3.CD36.CD38.CDK12.CDK13.CDK14.CDK4.CDKN1B.CELF5.CERS3.CERS5.CFLAR.CHD3.CHD5.CHKA.CHMP4C.CLN8.CLSPN.COMMD10.CPD.CPM.CPSF7.CREB1.CREB5.CROT.CSF2RA.CSRNP3.CTC1.CTH.CTSB.CYCS.CYLD.CYP3A4.DCAF10.DCAF5.DCAF6.DCN.DDOST.DDX51.DEGS1.DENND4A.DFFA.DHTKD1.DHX36.DIMT1.DIO2.DKC1.DLX1.DNPEP.DTX4.DUOX2.DUSP13.DUXA.DYRK1A.E2F3.EGR2.EIF2A.EIF4B.EIF4E.EIF4EBP2.EIF5.ELAVL1.ELK1.ELOVL3.ENSG00000173575.ENTPD1.EP300.EPHA6.EPHB2.ERCC1.EREG.ERLIN2.ERN2.ESCO2.ETF1.EZH1.FAM208A.FAM63B.FBXL5.FBXO6.FBXO9.FBXW11.FGFRL1.FKBP14.FKBP1B.FMO2.FOXD3.FOXK2.FOXO4.FOXP1.FSHB.FTO.FUT1.GALNT1.GALNT6.GAN.GATAD2B.GCM2.GDPD1.GEMIN8.GFPT1.GGT6.GIGYF2.GINS1.GLUD1.GLYCTK.GOLGA7.GOLGA7B.GPAM.GPD1L.GRAP.GRHL2.GRIN2B.GTF2H5.GTF2IRD2.GTF2IRD2B.H6PD.HCFC1.HDAC8.HEY2.HNRNPH3.HOXA2.HOXA9.HPSE2.HSP90B1.IBA57.ICMT.IGF2.IKZF2.ING4.IRAK1.IRAK2.ISCU.ITIH5.ITIH6.KALRN.KCTD9.KDM5B.KLF12.KLHDC7A.KMT2A.KMT2D.KPNA1.KSR2.LACTB.LARP1.LARP7.LARS.LDLR.LIMD1.LIN52.MAMLD1.MAP4K4.MAPK13.MAPT.MARC2.MARCH6.MBD6.MBTD1.MDM4.MECP2.METAP1.METTL2A.MGAT2.MGAT3.MGRN1.MGST2.MIXL1.MLXIP.MMP13.MMP14.MMP16.MRPL27.MRPL36.MRPL4.MRPL57.MTA3.MTMR4.MUC4.MYB.MYLK3.MYOCD.MZF1.NAF1.NCOR1.NDUFA4.NEDD8.NEIL1.NEK2.NEUROD6.NFAT5.NLK.NLN.NME6.NMNAT1.NMNAT2.NMT2.NOS1.NOVA2.NPAT.NR1D2.NR2F2.NTRK2.NWD1.OLIG2.ONECUT2.OTUD3.OTUD7B.P2RX7.PA2G4.PAFAH1B1.PAK3.PAN2.PANK1.PAPPA.PARP16.PATZ1.PAX5.PCYOX1.PCYT1A.PCYT1B.PDE4C.PDE7A.PDIA6.PDK4.PDP1.PEA15.PERP.PGPEP1.PHF3.PIAS2.PIK3R1.PIPOX.PKMYT1.PLAG1.PLOD1.PNPO.POLDIP3.POLH.POLR2E.POU2F1.POU3F2.PPARGC1A.PPM1K.PPP2R2A.PRDM11.PRKCA.PRKCB.PSAPL1.PSMB6.PTAR1.PTCH1.PTGIS.PTK6.PTP4A1.PTPRN2.PURA.PYCRL.RAB11A.RAB12.RAB3B.RAB7A.RAD23B.RAD51.RAD54L.RANBP9.RBL1.RC3H1.RHBDD1.RIOK2.RNASEH1.RNF135.RNF141.RNF146.RNF165.RORB.RPP14.RPRD2.RQCD1.RRM2.RRP1B.RUNX3.SAMD8.SAMHD1.SAP30L.SBF1.SCD5.SCRN3.SDF2.SDHD.SENP6.SEPHS2.SETD5.SETD7.SGMS1.SIN3A.SKP1.SLA.SLC19A3.SLC25A15.SLC35D1.SMAD4.SMARCC2.SMC3.SNRPD3.SOCS7.SP1.SP5.SPIB.SPOPL.SPTLC2.SPTLC3.SREK1.SREK1IP1.SRSF2.SRSF3.SSX1.ST3GAL3.ST6GAL1.ST6GAL2.STAT4.STK39.STOX1.STYXL1.SUFU.SUMO1.SUV39H2.TADA1.TAF5L.TBX15.TEAD1.TEK.TESK2.TET3.TEX2.TGFBR1.TGIF2.THUMPD3.TIMM50.TLK2.TMEM199.TMPRSS11A.TMX3.TNC.TNF.TP53.	GO

			TP53INP1.TP53INP2.TRABD2B.TRAF6.TRIM72.TRMT12.TRNAU1AP.TRPM7.TSR1.TTLL12.UBE2E3.UBE2J1.UBE3B.UBXN2B.ULK2.UNKL.UPF1.USB1.USP13.USP31.USP49.UST.UTP14C.VAPA.VEZF1.VHL.VIPAS39.WDR36.WTAP.XBP1.XPNPEP3.XXYLT1.ZBTB20.ZBTB4.ZBTB7A.ZBTB8A.ZDHHC22.ZDHHC23.ZEB1.ZFP14.ZFP30.ZFP91.ZFX.ZHX2.ZHX3.ZKSCAN1.ZMAT2.ZMYND8.ZNF131.ZNF160.ZNF182.ZNF189.ZNF229.ZNF25.ZNF317.ZNF331.ZNF335.ZNF33A.ZNF347.ZNF365.ZNF37A.ZNF426.ZNF430.ZNF440.ZNF445.ZNF454.ZNF460.ZNF483.ZNF486.ZNF490.ZNF500.ZNF514.ZNF555.ZNF557.ZNF568.ZNF592.ZNF625.ZNF696.ZNF700.ZNF711.ZNF773.ZNF791.ZNF793.ZNF805.ZNF84.ZNF865.ZYG11A	
DNA-binding transcription factor activity	125/1749	0.0102	ATF5.ATOH8.BACH2.BHLHE40.BLZF1.CAMTA1.CBL.CERS3.CREB1.CREB5.CSRNP3.DLX1.DLX6.DUXA.E2F3.EGR2.ELK1.EP300.EZH1.FOXD3.FOXK2.FOXO4.FOXP1.GATAD1.GATAD2B.GCM2.GRHL2.GTF2IRD2.GTF2IRD2B.HCFC1.HEY2.HOXA2.HOXA9.IKZF2.KDM5B.KLF12.KMT2A.KMT2D.MECP2.MIXL1.MLXIP.MTA3.MYB.MYOCD.MZF1.NCOR1.NEUROD6.NFAT5.NKX24.NR1D2.NR2F2.OLIG2.ONECUT2.PA2G4.PATZ1.PAX5.PLAG1.POU2F1.POU3F2.PURA.RORB.RUNX3.SIN3A.SMAD4.SMARCC2.SP1.SP5.SPIB.STAT4.TAF5L.TBX15.TEAD1.TET3.TGIF2.THAP8.TP53.UNKL.VEZF1.XBP1.ZBTB20.ZBTB4.ZBTB7A.ZEB1.ZFP14.ZFP30.ZFP91.ZFX.ZHX2.ZHX3.ZKSCAN1.ZMYM4.ZNF131.ZNF160.ZNF182.ZNF189.ZNF229.ZNF25.ZNF317.ZNF331.ZNF335.ZNF33A.ZNF347.ZNF37A.ZNF426.ZNF430.ZNF440.ZNF445.ZNF454.ZNF460.ZNF483.ZNF490.ZNF500.ZNF514.ZNF555.ZNF557.ZNF568.ZNF592.ZNF625.ZNF696.ZNF700.ZNF711.ZNF791.ZNF793.ZNF805.ZNF84	GO
Generic Transcription Pathway	93/ 1112	0.0024	AIFM2.BARD1.BDNF.C12orf5.CAMK2G.CAMK4.CBX2.CBX3.CCND2.CDK12.CDK13.CDK4.CDKN1B.CHD3.CREB1.CYCS.DLX6.EP300.GATAD2B.GPAM.GRIN2B.GTF2H5.HEY2.IRAK1.KDM5B.KMT2A.KMT2D.MAMLD1.MDM4.MMP13.MYB.NCOR1.NDUFA4.NR1D2.PAX5.PERP.POLR2E.PPARGC1A.PPP1R13B.PRKCB.PSMB6.RBL1.RORB.RQCD1.RRM2.RUNX3.SIN3A.SKP1.SMAD4.SMARCC2.SMARCD1.SP1.SUMO1.TEAD1.TGIF2.TNRC6A.TNRC6B.TNRC6C.TP53.TP53INP1.VEGFA.ZFP14.ZFP30.ZKSCAN1.ZNF160.ZNF189.ZNF25.ZNF317.ZNF331.ZNF33A.ZNF347.ZNF37A.ZNF426.ZNF430.ZNF440.ZNF445.ZNF454.ZNF460.ZNF483.ZNF486.ZNF490.ZNF500.ZNF514.ZNF555.ZNF557.ZNF568.ZNF625.ZNF696.ZNF700.ZNF711.ZNF773.ZNF791.ZNF793	Reactome
RNA Polymerase II Transcription	100/1233	0.0024	AIFM2.BARD1.BDNF.C12orf5.CAMK2G.CAMK4.CBX2.CBX3.CCND2.CDK12.CDK13.CDK4.CDKN1B.CHD3.CPSF7.CREB1.CYCS.DLX6.EP300.GATAD2B.GPAM.GRIN2B.GTF2H5.HEY2.IRAK1.KDM5B.KMT2A.KMT2D.MAMLD1.MDM4.MMP13.MYB.NCOR1.NDUFA4.NR1D2.PAX5.PERP.POLDIP3.POLR2E.POU2F1.PPARGC1A.PPP1R13B.PRKCB.PSMB6.RBL1.RORB.RPRD2.RQCD1.RRM2.RUNX3.SIN3A.SKP1.SMAD4.SMARCC2.SMARCD1.SNRPD3.SP1.SRSF2.SRSF3.SUMO1.TEAD1.TGIF2.TNRC6A.TNRC6B.TNRC6C.TP53.TP53INP1.VEGFA.ZFP14.ZFP30.ZKSCAN1.ZNF160.ZNF189.ZNF25.ZNF317.ZNF331.ZNF33A.ZNF347.ZNF37A.ZNF426.ZNF430.ZNF440.ZNF445.ZNF454.ZNF460.ZNF483.ZNF486.ZNF490.ZNF500.ZNF514.ZNF555.ZNF557.ZNF568.ZNF625.ZNF696.ZNF700.ZNF711.ZNF773.ZNF791.ZNF793	Reactome
Gene expression (Transcription)	106/1366	0.0034	AIFM2.BARD1.BDNF.C12orf5.CAMK2G.CAMK4.CBX2.CBX3.CCND2.CDK12.CDK13.CDK4.CDKN1B.CHD3.CPSF7.CREB1.CYCS.DLX6.EP300.GATAD2B.GPAM.GRIN2B.GTF2H5.HEY2.IRAK1.KDM5B.KMT2A.KMT2D.MAMLD1.MDM4.MMP13.MTA3.MYB.NCOR1.NDC1.NDUFA4.NR1D2.PAX5.PERP.POLDIP3.POLR2E.POM121.POM121C.POU2F1.PPARGC1A.PPP1R13B.PRKCB.PSMB6.RBL1.RORB.RPRD2.RQCD1.RRM2.RUNX3.SAP30L.SIN3A.SKP1.SMAD4.SMARCC2.SMARCD1.SNRPD3.SP1.SRSF2.SRSF3.SUMO1.TEAD1.TET3.TGIF2.TNRC6A.TNRC6B.TNRC6C.TP53.TP53INP1.VEGFA.ZFP14.ZFP30.ZKSCAN1.ZNF160.ZNF189.ZNF25.ZNF317.ZNF331.ZNF33A.ZNF347.ZNF37A.ZNF426.ZNF430.ZNF440.ZNF445.ZNF454.ZNF460.ZNF483.ZNF486.ZNF490.ZNF500.ZNF514.ZNF555.ZNF557.ZNF568.ZNF625.ZNF696.ZNF700.ZNF711.ZNF773.ZNF791.ZNF793	Reactome

FDR. false discovery rate; GO. gene ontology: KW. keyword.
